# Cyclofaulknamycin with the Rare Amino Acid D-capreomycidine Isolated from a Well-Characterized *Streptomyces albus* Strain

**DOI:** 10.3390/microorganisms9081609

**Published:** 2021-07-28

**Authors:** Liliya Horbal, Marc Stierhof, Anja Palusczak, Nikolas Eckert, Josef Zapp, Andriy Luzhetskyy

**Affiliations:** 1 Department of Pharmaceutical Biotechnology, Saarland University, 66123 Saarbruecken, Germany; lihorbal@gmail.com (L.H.); marc.stierhof@uni-saarland.de (M.S.); anja.palusczak@uni-saarland.de (A.P.); 2 AMEG Department, Helmholtz Institute for Pharmaceutical Research Saarland, 66123 Saarbruecken, Germany; nikolas.eckert@uni-saarland.de; 3 Department of Pharmaceutical Biology, Saarland University, 66123 Saarbruecken, Germany; j.zapp@mx.uni-saarland.de

**Keywords:** D-capreomycidine, cyclofaulknamycin, cyclopeptide, *Streptomyces*

## Abstract

Targeted genome mining is an efficient method of biosynthetic gene cluster prioritization within constantly growing genome databases. Using two capreomycidine biosynthesis genes, alpha-ketoglutarate-dependent arginine beta-hydroxylase and pyridoxal-phosphate-dependent aminotransferase, we identified two types of clusters: one type containing both genes involved in the biosynthesis of the abovementioned moiety, and other clusters including only arginine hydroxylase. Detailed analysis of one of the clusters, the *flk* cluster from *Streptomyces albus*, led to the identification of a cyclic peptide that contains a rare D-capreomycidine moiety for the first time. The absence of the pyridoxal-phosphate-dependent aminotransferase gene in the *flk* cluster is compensated by the *XNR_1347* gene in the *S. albus* genome, whose product is responsible for biosynthesis of the abovementioned nonproteinogenic amino acid. Herein, we report the structure of cyclofaulknamycin and the characteristics of its biosynthetic gene cluster, biosynthesis and bioactivity profile.

## 1. Introduction

Cyclic peptides possess a wide variety of biological activities with high application potentials as antibiotics (e.g., vancomycin and teicoplanin) and phytopathogenic agents (e.g., cyclothiazomycin, neopeptin, and kutznerides) [1,2,3]. Their biosynthesis is accomplished via ribosomal [4] or nonribosomal biosynthetic machinery [5]. Peptides of both origins show extremely high structural diversity resulting from the presence of a large variety of nonproteinogenic amino acids that are either used for the biosynthesis of the peptides or generated as a result of post-translational modifications [4,6]. A growing group of peptide compounds with important biological activities contains a rare structural class of amino acids, derivatives of arginine with unique five- or six-membered cyclic guanidine moieties (enduracididine and capreomycidine, respectively) [7,8]. For instance, mannopeptimycin and its semisynthetic analogs represent a new class of lipoglycopeptides with exceptional activity against MRSA, VRE, and penicillin-resistant *Streptococcus pneumoniae* [9,10]. Enduracidins A and B are depsipeptides containing two enduracididine moieties that are active against Gram-positive bacteria, including resistant strains and *Mycobacterium* species [11]. Teixobactin is a peptide-like compound with potent activity against different multidrug-resistant bacteria, including *M. tuberculosis* and *Clostridium difficile* [12], and does not induce the development of resistance [13]. Viomycin, which is an essential component in the drug cocktail currently used to treat *M. tuberculosis* infections, also contains the capreomycidine amino acid moiety [14]. A broad spectrum of evidence supports the notion that the presence of arginine residues in linear or cyclic antimicrobial peptides enhances their activity [15]. For example, the relevance of arginine to the activity of small peptides was demonstrated by less potent lysine-containing analogs of an 11-residue lactoferricin segment [16]. In addition to peptide compounds, other classes of natural products (NPs) containing arginine residues, or derivatives thereof, possess promising activities [8,17]. Recently, it was shown that the incorporation of arginine residue into the chemical structure of vancomycin confers it with cell-killing activity against carbapenem-resistant *E. coli*, which are Gram-negative bacteria [18]. Thus, NPs containing rare nonproteinogenic amino acid derivatives, including arginine, and their coding gene clusters, are of particular interest because they can serve as chemical scaffolds for biochemical and semisynthetic modifications to expand their biochemical diversity and as a source of genes for combinatorial biosynthesis. These features make them an important source of pharmacologically active and industrially relevant secondary metabolites.

In this article, we describe the utilization of two genes encoding enzymes responsible for the biosynthesis of the capreomycidine moiety, alpha-ketoglutarate-dependent arginine beta-hydroxylase, and pyridoxal-phosphate-dependent aminotransferase, for the targeted genome mining of new peptide compounds containing the abovementioned amino acid. The genome analysis of a well-characterized *S. albus* strain revealed the presence of an arginine beta-hydroxylase-encoding gene homolog in one of the NRPS gene clusters. To identify the product of this cluster, the metabolomes of two previously developed *S. albus* mutant strains [19] with and without the abovementioned cluster were compared. The analysis and comparison of the secondary metabolite profiles of the two strains allowed us to identify compounds with molecular masses of 731.4 and 749.4 Da, which correspond to the molecular weights of the predicted but never-identified cyclic faulknamycin compound and the recently described linear faulknamycin, respectively [20]. This is the first identified cyclic peptide compound with the rare D-capreomycidine amino acid. Furthermore, a gene encoding pyridoxal-phosphate-dependent aminotransferase located outside of the identified cluster is shown to be involved in the biosynthesis of the D-capreomycidine moiety. Herein, we report the structure of this compound and describe its biosynthetic gene cluster, biosynthesis and bioactivity profile.

## 2. Materials and Methods

### 2.1. Bacterial Strains and Culture Conditions

The bacterial strains used in this study are listed in Table 1. The *E. coli* strains were grown in Luria–Bertani (LB) broth medium. When required, antibiotics (Carl Roth, Karlsruhe, Germany; Sigma-Aldrich, St. Louis, MO, USA) were added to the cultures at the following concentrations: 75 μg mL^−1^ ampicillin, 50 μg mL^−1^ kanamycin, 50 or 120 μg mL^−1^ hygromycin, and 50 μg mL^−1^ apramycin (Carl Roth, Karlsruhe, Germany; Sigma-Aldrich, St. Louis, MO, USA). *E. coli* GB05-red [21] was employed in Red/ET recombineering experiments [22].

For conjugation, the *Streptomyces albus* J1074, del9 and del10 [19] strains were grown on oatmeal or mannitol soy (MS) agar [23] for sporulation.

### 2.2. Recombinant DNA Techniques

Chromosomal DNA from *Streptomyces* strains and plasmid DNA from *E. coli* were isolated using standard protocols [23,24]. Restriction enzymes and molecular biology reagents were used as per the manufacturer’s protocol (NEB, Ipswich, UK; Thermo Fisher Scientific, Waltham, MA, USA).

### 2.3. Construction of the delXNR_1347 BAC Vector

In the Red/ET recombination experiment, a linear DNA fragment containing an apramycin resistance marker and an origin of transfer (*oriT*) flanked by suitable homology arms was generated by PCR using the XNR_1347RedFor and XNR_1347RedRev primer pair (Table 2). PCR was carried out with Phusion DNA polymerase (Thermo Fisher Scientific, Waltham, MA, USA), according to the manufacturer’s protocol. The PCR product was concentrated by ethanol precipitation prior to further use. In general, 300 µL of an overnight culture of *E. coli* GB05-red (Table 1) cells harboring the parent to be modified, 2K9/2 BAC, was inoculated into 15 mL of LB, and the culture was incubated on a shaker at 37 °C and 200 rpm for 2 h. Thereafter, 400 µL of 10% L-rhamnose was added to the culture to induce the expression of the recombinases. Cultivation was then continued for 45 min. The cells were subsequently harvested by centrifugation, washed twice with ice-cold distilled H_2_O, and resuspended in 600 µL of 10% ice-cold glycerol. The PCR product was mixed with the electrocompetent cells, which were then transferred to an ice-cold electroporation cuvette (1 mm). The mixture was subsequently electroporated at 1800 V (Eppendorf electroporator), followed by the addition of 750 µL LB. The cells were cultivated at 37 °C for 90 min before the culture was transferred to LB agar plates with apramycin. The plates were incubated at 37 °C overnight. Correct transformants were verified by the restriction analysis and sequencing of the isolated BAC DNA using the XNR_1347F and XNR_1347R primers (Table 2).

### 2.4. Construction of the S. albus delXNR_1347 Mutant

The 2K9/2delXNR_1347 (Table 1) gene disruption BAC was transferred from *E. coli* ET12567/pUB307 cells into *S. albus* del9 cells by means of conjugation [23]. Transconjugants were selected for resistance to apramycin (50 μg mL^−1^) and glucuronidase activity. The abovementioned cosmid did not contain the origin of replication for *Streptomycetes*, all the obtained exconjugants were the result of a first crossover event. For the generation of the *S. albus* ΔXNR_1347 mutant, single-crossover apramycin and blue mutants were screened for the loss of glucuronidase activity (blue pigmentation) as the result of a double-crossover event. The replacement of the XNR_1347 gene was confirmed by PCR using the XNR_1347F and XNR_1347R primer pair (Table 2).

### 2.5. Metabolite Extraction and Analysis

*S. albus* strains were cultivated in 25 mL TSB medium for 48 h at 28 °C. The main cultures containing 50 mL of SG were inoculated with 2 mL of preculture. After 5 days of cultivation at 28 °C, the secreted metabolites were extracted with ethyl acetate and butanol, followed by solvent evaporation. The dry extracts were dissolved in 0.5 mL methanol, and 1 μL of the dissolved sample was separated in a Dionex Ultimate 3000 RSLC system using a BEH C18, 100 × 2.1 mm, 1.7 μm dp column (Waters Corporation, Milford, MA, USA). The separation of a 1 μl sample was achieved via a linear gradient with a mobile phase of water/acetonitrile, each containing 0.1% formic acid, with a gradient from 5–95% acetonitrile applied over 9 min at a flow rate of 0.6 mL/min and 45 °C. High-resolution mass spectrometry was performed on an Accela UPLC system (Thermo Fisher Scientific, Waltham, MA, USA) coupled to a LTQ Orbitrap XL mass spectrometer ( Thermo Fisher Scientific, Waltham, MA, USA) operating in positive or negative ionization modes. Data were collected and analyzed with Thermo Xcalibur software, version 3.0 (Thermo Scientific, Waltham, MA, USA). The monoisotopic mass was searched in a natural product database.

### 2.6. Isolation and Purification of Cyclic and Linear Iso-Faulknamycin

For faulknamycin production, 2 mL of a 2-day-old preculture grown in 50 mL of TSB media (Sigma-Aldrich, St. Louis, MO, USA) was inoculated into 100 mL of SG media, and the culture was grown for 5 days at 28 °C with agitation at 200 rpm. The methanol extract obtained after 10 L butanol extraction was used for the purification of the compounds in an IsoleraTM One flash purification system (Biotage, Uppsala, Sweden) equipped with a CHROMABOND Flash RS 330 C18 ec column (Macherey-Nagel, Dueren, Germany) using a gradient of 5–95% aqueous methanol for 3 CV at a flow rate of 100 mL/min, with UV detection at 210 and 280 nm. The fractions containing both faulknamycin compounds were collected, pooled together, dried and dissolved in methanol. Thereafter, size-exclusion chromatography was performed on an LH20 Sephadex column (Sigma-Aldrich, Louis, MO, USA). Methanol was used as a solvent for elution. Fractions were collected every 12 min at a flow rate of 0.6 mL/min. The fractions containing faulknamycins were pooled together, evaporated, and dissolved in methanol. Then, the prepurified methanol extract was further separated by preparative reversed-phase (RP) HPLC (Waters 2545 Binary Gradient module, Waters, Milford, MA, USA) using a Nucleodur C18 HTec column (5 μm, 21 × 250 mm, Macherey Nagel, Dueren Germany) with a linear gradient of 0.1% formic acid solution in acetonitrile against 0.1% formic acid solution in water, applied at 5% to 95% over 33 min at a flow rate of 5 mL/min. UV spectra were recorded with a PAD detector (Photodiode Array Detector, Waters, Milford, MA, USA). The fractions containing the linear and cyclic faulknamycin compounds were dried and dissolved in methanol. The final purification step was performed in an RP HPLC system (Agilent Infinity 1200 series HPLC system) equipped with a SynergiTM 4 µm Fusion-RP 80 Å 250 × 10 (Phenomenex, Torrance, CA, USA) column using a linear gradient of [A] H^2^O + 0.1% formic acid/[B] acetonitrile + 0.1% formic acid, applied at 5% to 95% [B] over 25 min at a flow rate of 3 mL/min, with a column oven temperature of 45 °C and UV detection at 210 nm, followed by fraction control via HPLC-MS. Fractions were pooled to obtain the two pure faulknamycin isolates (1.2 mg of linear faulknamycin and 1.4 mg of cyclic faulknamycin).

### 2.7. Nuclear Molecular Resonance Spectroscopy (NMR)

NMR spectra were acquired on a Bruker Avance III, Ascent 700 MHz spectrometer (298 K) equipped with a 5 mm TCI cryoprobe (Bruker, BioSpin GmbH, Rheinstetten, Germany). The chemical shifts (δ) were reported in parts per million (ppm) relative to TMS. As a solvent, deuterated DMSO-d6 (δH 2.50 ppm., δC 39.51 ppm.) from Deutero (Kastellaun, Germany) was used. Edited-HSQC, HMBC, 1H-1H COSY and ROESY spectra were recorded using the standard pulse programs from Bruker TOPSPIN v.3.6 software.

### 2.8. Marfey’s Method

Iso-faulknamycin, chymostatin and capreomycin were hydrolyzed in 100 µL 6 N HCl at 110 °C for 45 min. While cooling down, the samples were dried for 15 min under nitrogen and dissolved in 110 mL water, after which 50 µL of each sample was transferred to a 1.5 mL Eppendorf tube. Next, 20 µL of 1 N NaHCO_3_ and 20 µL of 1% L-FDLA or D-FDLA in acetone were added to the hydrolysates. The amino acid standards were prepared in the same way using only L-FDLA. The reaction mixtures were incubated at 40 °C for 90 min at 750 rpm and subsequently quenched with 2 N HCl to stop the reaction. The samples were diluted with 300 µL ACN, and 1 µL of each sample was analyzed in a maXis high-resolution LC-QTOF system using aqueous ACN with 0.1 vol% FA and an adjusted gradient of 5–10 vol% for 2 min, 10–25 vol% for 13 min, 25–50 vol% for 7 min and 50–95 vol% for 2 min. Sample detection was carried out at 340 nm.

### 2.9. Genome Mining and Bioinformatic Analysis

Genome screening was performed using the BLAST online tool (blast.ncbi.nlm.nih.gov/Blast.cgi (accessed on 15 June 2021)). The identified genomes were downloaded from the NCBI genome database (www.ncbi.nlm.nih.gov/genome/ (accessed on 15 June 2021)). Gene cluster analysis was performed using the antiSMASH online tool (antismash.secondarymetabolites.org/#!/start (accessed on 15 June 2021)) [25]. Analysis of genetic data was performed using Geneious software, version 11.0.3 (Biomatters Ltd., Auckland, New Zealand).

### 2.10. Antimicrobial Susceptibility Test

Minimum inhibitory concentrations (MICs) were determined according to standard procedures. Single colonies of the bacterial strains were suspended in cation-adjusted Müller-Hinton broth to achieve a final inoculum of approximately 104 CFU mL^−1^. Serial dilutions of cyclofaulknamycin (0.03 to 64 µg·mL^−1^) were prepared in sterile 96-well plates, and the bacterial suspension was added. Growth inhibition was assessed after overnight incubation (16–18 h) at 30–37 °C. A panel consisting of the following microbial strains was tested: *Acinetobacter baumannii* DSM-30008, *E. coli* JW0451-2 (ΔacrB), *S. aureus* Newman, *E. coli* BW25113 (wt), *M. smegmatis* mc2155, *P. aeruginosa* PA14, *B. subtilis* DSM-10, *Citrobacter freundii* DSM-30039, *Mucor hiemalis* DSM-2656, *Candida albicans* DSM-1665, *Cryptococcus neoformans* DSM-11959, and *Pichia anomala* DSM-6766.

## 3. Results and Discussion

### 3.1. Mining of Actinobacterial Genomes for the Presence of Capreomycidine Biosynthetic Genes

**Figure 1:**
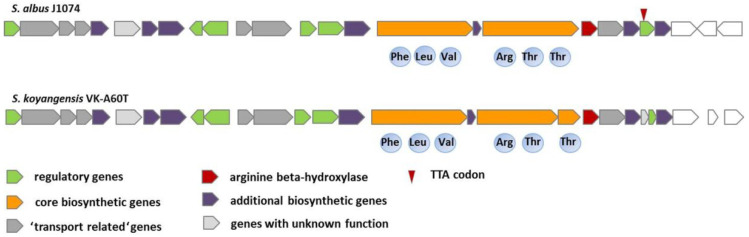
Structure of the *flk* clusters in the *S. albus* and *S. koyangensis* strains.

To identify the compounds containing the rare cyclic guanidino-amino acid capreomycidine, two genes, *vioC* (encoding alpha-ketoglutarate-dependent arginine beta-hydroxylase) and *vioD* (encoding the PLP-dependent aminotransferase (PLP-aminotransferase) from the viomycin biosynthetic gene cluster [26], were used for the targeted genome analysis and cluster searches in the NCBI database. As a result of the in silico mining performed, two types of biosynthetic gene clusters (BGCs) were identified: one that contained both homologs, and other clusters that included only one of the two genes (the *vioC* homolog). We focused our attention on the second group of clusters, which have not previously been characterized. Until recently, the products of both gene orthologs (VioC and VioD) were considered to be required for capreomycidine biosynthesis [26]. During the preparation of this manuscript, an article describing the new hypothetical pathway for capreomycidine biosynthesis was published [20]. From the identified clusters, two *flk* BGCs that had a very similar structure and NRPS domain organization attracted our attention; however, they were identified in different microorganisms, *S. albus* J1074 and *Streptomyces koyangensis* VK-A60T (Figure 1).

Furthermore, the recently published faulknamycin gene cluster (fau BGC) [20] appeared to be very similar to the *flk* BGCs. Based on adenylation domain analysis, the two clusters were predicted to encode hexapeptides composed of the same amino acids (Figure 1). The main difference between the two identified BGCs was the presence of two and three NRPS genes in the *S. albus* J1074 and *S. koyangensis* VK-A60T clusters, respectively (Figure 1). PLP-dependent aminotransferase genes were not present in these clusters or in the recently identified faulknamycin gene cluster. Tryon et al. suggested that the capreomycidine moiety might be synthesized via the epimerization domain of the FauG NRPS. However, at least one homolog of the PLP-aminotransferase gene was identified within the *S. albus* J1074 (XNR_1347) and *S. koyangensis* VK-A60T genomes after careful manual analysis. Taking into account this information, it was assumed that the XNR_1347 product might be involved in the biosynthesis of the capreomycidine moiety. The chemical products of the flk clusters were not known; however, they were assumed to be identical to faulknamycin based on the in silico prediction of the NRPS domain organization and the genes present in the clusters (Figure 1).

### 3.2. Analysis of an flk Gene Cluster in the S. albus Genome

The product of biosynthetic gene cluster 5 (*flk* cluster) from the well-studied *S. albus* strain has never been described before. Based on in silico analysis and comparison with a similar cluster from the *S. koyangensis* VK-A60T strain (Table S1, Figure 1), it was assumed that the *flk* cluster contained 23 genes encoding proteins that are sufficient for final peptide biosynthesis (Figure 1, Table S1). Among these genes, *flkP* and *flkS* encode two large multifunctional NRPSs (Figure 1, Table S1) that are composed of six biosynthetic modules and are therefore assumed to encode hexapeptides. Detailed analysis of the NRPSs, with the help of NRPS Predictor 2 (abi-services.informatik.informati/tuebingen.de/nrps2/Controller?cmd=SubmitJob (accessed on 15 June 2021)), allowed us to identify the adenylation domain (A-domain) specificity of the individual modules (Table 3). Based on the substrate specificity-conferring codes, it was assumed that domain A1 was responsible for the incorporation of phenylalanine, A3 for valine, and A5 and A6 for threonine. The remaining two domains, A2 and A4, are characterized by substrate promiscuity; however, they were predicted to most likely be responsible for the incorporation of leucine and arginine or its derivatives, respectively (Table 3). Sequence analysis of the FlkP NRPS revealed that it contained the following domains: three A domains, three peptidyl carrier protein (PCP) domains, one epimerization domain and two condensation domains. These domains form three modules, including one loading module and two elongation modules, with the following domain organization: A_Phe_-PCP-C-A_Leu_-PCP-E-C-A_Val_-PCP. E represents an epimerization domain which is responsible for the conversion of L-amino acids into the D-form. The FlkP protein ends with a PCP domain; thus, its product should be offloaded to the next multidomain NRPS enzyme, which is FlkS. FlkS also contains three modules: C-A_Arg_-PCP-E-C-A_Thr_-PCP-C-A_Thr_-PCP-E. The presence of the two epimerization domains means that two D-amino acids are incorporated into the peptide during its biosynthesis. NRPS chain-terminating thioesterases are often found to exist as terminating domains in modular NRPSs [27]. However, no such domain was detected in the FlkP or FlkS proteins. Only the type II thioesterase homolog FlkX was identified in the *flk* cluster as a stand-alone gene, whose product is assumed to show proofreading activity. A homolog of the MppK alpha/beta hydrolase from the mannopeptimycin gene cluster, FlkO, was identified in the cluster and is assumed to be involved in the release of the hexapeptide from the biosynthetic machinery and its further cyclization. The *flkR* gene is located between two NRPS genes and encodes an MbtH homolog which is suggested to be involved in the promotion of NRPS production [28]. *FlkT* is a homolog of *vioC* [7] and encodes Fe(II)/alpha-ketoglutarate-dependent arginine beta-hydroxylase, which is involved in the biosynthesis of the capreomycidine moiety [26]. No homolog of the PLP-dependent aminotransferase is present in the *flk* cluster or in the cluster from *S. koyangensis*. Using BLAST analysis, we identified an *XNR_1347* gene encoding a PLP-dependent aminotransferase located outside of the *flk* gene cluster, which might be involved in capreomycidine biosynthesis. Another possibility is that the biosynthesis of the capreomycidine moiety is ongoing, as suggested by Tryon et al., 2020. *FlkG* and *flkH* encode methyl- and mannosyltransferases, respectively, and might be involved in post-translational modifications of the core peptide. Homologs of these genes are also present in the mannopeptimycin biosynthetic gene cluster [10].

Two stand-alone putative FlkA and FlkW regulators, which belong to the DeoR/GlpR and ArsR families, respectively, are present in the cluster and might be involved in the regulation of biosynthesis. Furthermore, FlkW contains a TTA codon in the coding frame and is thus subject to *bldA* regulation [29]. Two pairs of histidine kinases and response regulators (FlkJ/FlkI and FlkN/FlkM) were detected in the cluster and might therefore also be involved in the regulation of gene expression.

Three genes, *flkB*, *flkV* and *flkD*, encode putative sugar transporters that might be involved in sugar uptake pathways. Transporters with similar putative functions (MppE and F) are also present in the mannopeptimycin gene cluster [10]. *FlkK* and *flkL* encode ABC transporters, and *flkU* encodes a major facilitator superfamily transporter and might thus be involved in antibiotic resistance.

### 3.3. Identification of the flk Cluster Product

Although *S. albus* has been subjected to a great deal of investigation as a model *Streptomyces* strain, the product of the *flk* cluster remains unknown. To identify this product, we took advantage of two constructed mutant strains, *S. albus* del9 and del10, published by Myronovsyki et al., 2018. The *S. albus* del9 strain contains the *flk* gene cluster, whereas del10 is characterized by the deletion of this cluster [19]. Both strains were cultivated in SG production medium for 5 days, the culture broths were extracted with ethyl acetate and butanol separately, and the obtained extracts were analyzed using LC/MS (for details, see the Section 2). Comparison of the biosynthetic profiles of the two strains revealed the disappearance of the molecular ions [M + H+] with masses of 750.4 and 732.4 Da from the metabolic profile of the del10 strain (Figure S21). Thus, the *flk* cluster was considered to be responsible for the biosynthesis of peptides with the above molecular masses. A search for compounds with such masses in the dictionary of natural products did not reveal any hits; therefore, the products were considered to be new. However, during the preparation of this manuscript, a linear compound with one of these masses (750.4 Da), referred to as faulknamycin, was published [20].

### 3.4. Purification and Structural Elucidation of Iso- and Cyclofaulknamycins

To gain insight into the structures of the produced compounds, the producer strain *S. albus* del9 was cultivated in 10 L of SG production medium, and the metabolites were extracted from the culture supernatant with butanol. The compounds with masses of 749.4 and 731.4 Da were successfully purified from the extract.

**Figure 2:**
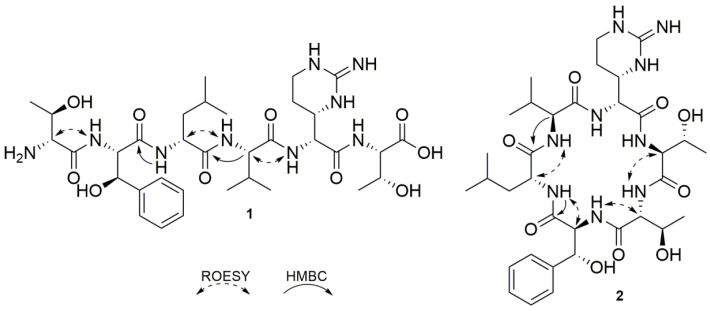
Structures of iso-faulknamycin (**1**) and cyclofaulknamycin (**2**) showing key HMBC and ROESY correlations.

The structure of iso-faulknamycin (1) (Figure 2) was established by NMR spectroscopy and MS/MS fragmentation. The molecular formula was determined to be C_34_H_55_N_9_O_10_ based on the identification of a mass of 749.40 Da and 12 degrees of unsaturation. In 2D NMR experiments (Figure S1, Table S2), including COSY (Figure S2), edited-HSQC (Figure S3) and HMBC (Figure S4) analyses, six amino acids assigned to leucine, valine, two threonines and two modified amino acids originating from phenylalanine and arginine were identified. The phenylalanine-derived unit showed HMBC correlations from its isochronic phenyl ring protons H-2 and H-6 (7.42, 2H) to a methine at δC 72.8 (Figure S4) in the side chain. Hence, it was assumed that a hydroxyl group in the β position resulted in the assignment of β phenylserine. Considering the four remaining unassigned nitrogens calculated from the molecular formula, it was concluded that one of the amino acids had to be related to arginine. Its α CH proton (δH 4.70) revealed a COSY correlation to a neighboring methine at δH 3.73 (Figure S2). The remaining two degrees of unsaturation indicated an intramolecular ring closure of the guanidinium moiety and the β-position, leading to the assignment of capreomycidine. The sample did not dissolve completely in DMSO-d6, which led to a lack of signal strength and prevented the full annotation of all carbon signals. However, by combining the observable HMBC and ROESY correlations, a major portion of the sequence could be assigned (Figure 2). The missing data were filled in by MS/MS fragmentation (Figures S17 and S18), revealing a final sequence of H2N Thr β-phenylserine-Leu-Val-capromycidine-Thr-COOH.

Based on known hexapeptide structures, which are mostly cyclic, it was assumed the compound with a mass of 731.39 Da with a calculated formula of C_34_H_53_N_9_O_9_ and 13 degrees of unsaturation represented the cyclic version of iso-faulknamycin 1. In comparison to 1, the observation of water loss and an additional degree of unsaturation indicated ring formation by intramolecular condensation. In 2D NMR experiments (Figures S6–S10, Table S3), it was possible to identify the same amino acids previously determined for iso-faulknamycin. Similar to linear hexapeptide 1, the cyclic version suffered from solubility issues, and the long-range HMBC and ROESY correlation had to be supported by MS/MS fragmentation (Figures S19 and S20). The cyclic formation of the new compound was confirmed by comparing the integral values of the NH signals and their ROESY correlations. The threonine NH_2_ group of the linear peptide showed a shift of δH 7.88 and an integral value of two protons, while the same signal of the cyclic version showed a low-field shift to δH 8.52 with an integral of only one proton. Furthermore, a ROESY correlation between this NH of the first threonine and the α-CH proton of the second threonine was identified, both of which were still terminal in linear peptide 1 but directly adjacent in cyclic peptide 2 (Figure 2). In addition, the MS/MS fragmentation of the [M + 2H]^2+^ ion revealed intense a-ion and x-ion fragments, which could only be observed for the cyclic peptide (Table S6). This allowed us to confirm the assignment of cyclic version 2, which was named cyclofaulknamycin.

Compounds 1 and 2 were named after a homologous structure that was recently published and designated faulknamycin [20]. The amino acid sequence and molecular formula of the abovementioned compound 1 appeared to be the same as those of faulknamycin. To determine whether 1 was identical to faulknamycin, the absolute stereochemistry of compound 1 was determined by Marfey’s method (see Supplementary Materials) [30]. The amino acid mixture was compared with commercially acquired standards derivatized with L-FDLA (Figure S11). The configuration of capreomycidine was determined as described previously by using standards derived from the hydrolysis of capreomycidine and chymostatin and derivatization by L-FDLA and D-FDLA [20] (Figure S13). The amino acid β-phenylserine has been identified by Tryon et al. as the D-*threo-* isomer; therefore, DL-*threo*-β-phenylserine was used as a standard to confirm the reported stereochemistry (Figures S14 and S15). This resulted in the identification of L-valine, D-leucine, L-threonine, D-*allo*-threonine, D-capreomycidine and L-*threo*-β-phenylserine as the constituent amino acids. D-*threo*-β-phenylserine could not be identified as a result of the performed analysis; thus, compound 1 was concluded to be an isomer of faulknamycin and was named iso-faulknamycin (Figure S16). This finding is in line with the domain organization of the FlkP NRPS described above.

### 3.5. Deletion of the XNR1347 Gene in the S. albus del9 Genome

**Figure 3:**
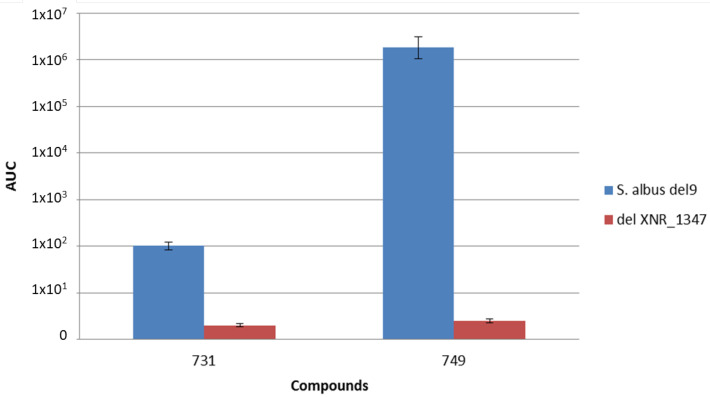
Faulknamycin production in the *S. albus* del9 and delXNR_1347 strains: 731–cyclofaulknamycin; 749–iso-faulknamycin.

Only one PLP-dependent aminotransferase-encoding gene, *XNR_1347*, has been found in the *S. albus* genome; therefore, it was assumed that its product might be involved in the biosynthesis of the capreomycidine amino acid. To test this hypothesis, the deletion of this gene was performed in the del9 strain. For this purpose, the *S. albus* BAC library was used [19]. The coding sequence of the *XNR_1347* gene in the 2K9/2 BAC was substituted with the apramycin resistance marker *aac(3)IV* and *oriT* via a Red/ET technology-based method (for details, see the Section 2), resulting in the construction of the 2K9/2delXNR_1347 deletion BAC (Table 2). The obtained construct was checked by restriction analysis as well as by partial sequencing. The cosmid was transferred into the *S. albus* del9 strain via conjugation. The obtained transconjugants were selected based on the resistance to apramycin and blue color, which denoted the presence of glucuronidase activity. Single-crossover mutants were screened for the loss of blue color resulting from a double-crossover event. As a result, the *S. albus* Δdel9delXNR_1347 strain (Table 1) carrying the *XNR_1347* gene deletion was created. The identity of the strain was confirmed by PCR analysis (data not shown). LC-MS analysis of the extracts from the *S. albus* Δdel9 and Δdel9delXNR_1347 strains (Figure 3) revealed 5- and 12-fold average decreases in the production of iso- and cyclofaulknamycins, respectively. Only traces of the compounds were detectable, which confirmed the hypothesis that the product of the *XNR_1347* gene is involved in the biosynthesis of the capreomycidine moiety. Furthermore, this seems to be the main mechanism of capreomycidine moiety formation in *S. albus*.

### 3.6. Proposed Biosynthesis of Cyclofaulknamycin

**Figure 4:**
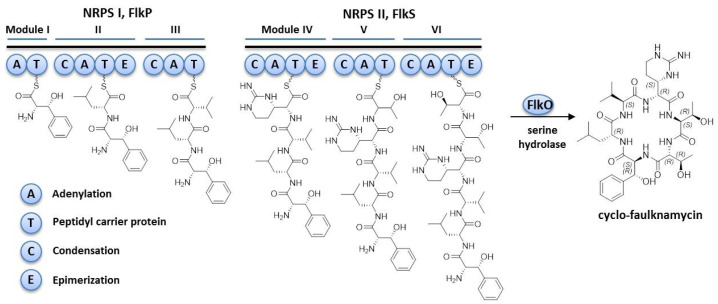
Proposed biosynthesis of cyclofaulknamycin.

Tryon et al. (2020) never detected cyclofaulknamycin. In the current study, however, the cyclofaulknamycin product of the *flk* cluster was isolated, and its structure was elucidated with the aid of NMR and MS2 experiments (for details, see the section above). A detailed analysis of NRPS domain organization and cyclofaulknamycin chemical structure, as well as a comparison of the gene products in the cluster with previously described enzymes, allowed us to propose its biosynthesis process (Figure 4). The FlkP domain, in contrast to FauE and FauG from the *fau* cluster, contains a clear loading module which is responsible for the incorporation of the first amino acid, phenylalanine, or possibly its derivative, hydroxyphenylalanine, and two elongation modules that are responsible for leucine and valine incorporation. The epimerization domain is responsible for the conversion of L-leucine into its D-form. FlkP ends with the peptidyl carrier protein; therefore, its product has to be offloaded to the next NRPS multidomain enzyme, FlkS. The first module of FlkS is responsible for the incorporation of the capreomycidine moiety, and the last two modules are responsible for the incorporation of two threonines. The biosynthesis of capreomycidine starts with the hydroxylation of arginine with the help of the VioC homolog Fe(II)/alpha-ketoglutarate-dependent arginine beta-hydroxylase FlkT. Thereafter, the XNR_1347 PLP-dependent aminotransferase catalyzes its conversion into capreomycidine. The deletion of *XNR_1347* did not lead to a complete cessation of biosynthesis; therefore, it was assumed that some other aminotransferase homologs in the genome might be responsible for the biosynthesis of this amino acid or that it occurs spontaneously, as described by Tryon et al. The presence of epimerization domains in the first two modules is responsible for the conversion of capreomycidine and threonine into their D-forms. No classical PKS/NRPS macrocyclizing thioesterase I is present in the cluster; thus, the serine hydrolase homolog FlkO is most likely responsible for the release and cyclization of faulknamycin.

### 3.7. Bioactivity Profile

Many known cyclic peptide NPs containing arginine derivatives, such as viomycin, mannopeptimycin, and capreomycin, are characterized by antibacterial activities [9,10]. Cyclofaulknamycin was identified in this study as a cyclic peptide and contains a capreomycidine moiety; therefore, it was assumed to possess some interesting activities. Thus, it was tested against a panel of bacterial, yeast and mold strains, including *Acinetobacter baumannii* DSM-30008, *E. coli* JW0451-2 (ΔacrB), *S. aureus* Newman, *E. coli* BW25113 (*wt*), *M. smegmatis* mc2155, *P. aeruginosa* PA14, *B. subtilis* DSM-10, *Citrobacter freundii* DSM-30039, *Mucor hiemalis* DSM-2656, *Candida albicans* DSM-1665, *Cryptococcus neoformans* DSM-11959, and *Pichia anomala* DSM-6766 (for details, see the Section 2). The tested concentrations were 0.03–64 µg·mL^−1^. However, no activity was detected against the tested strains in the abovementioned range (data not shown).

## 4. Conclusions

Sequence databases are a potential source of new chemical scaffolds which can be accessed by different prioritization approaches [31,32]. Herein, we applied targeted genome mining based on the utilization of alpha-ketoglutarate-dependent arginine beta-hydroxylase and pyridoxal-phosphate-dependent aminotransferase genes responsible for the biosynthetic formation of the capreomycidine moiety to identify new peptide NPs and their corresponding biosynthetic gene clusters. Using this approach, we identified two groups of clusters: one group contained both genes, and the other contained only the homolog encoding arginine beta-hydroxylase. The latter type of cluster was not known to exist until recently, because the products of both genes were considered to be required for the biosynthesis of L-capreomycidine [26]. Detailed analysis of one of these clusters, the *flk* cluster, in the genome of the model organism *S. albus* enabled us to identify a cyclic peptide containing D-capreomycidine (rather than L-capreomycidine, as commonly found) for the first time. Only linear faulknamycin has previously been described to contain a D-capreomycidine moiety [20]. The detailed analysis of the *flk* cluster allowed us to suggest the biosynthesis mechanisms of cyclofaulknamycin and its capreomycidine moiety (Figure 4). For the first time, a PLP-dependent aminotransferase gene product generated from a site located outside of the cluster was shown to be involved in the biosynthesis of the capreomycidine moiety of cyclofaulknamycin. However, cyclofaulknamycin biosynthesis was not completely blocked in the delXNR_1347 mutant, suggesting that other homologs of the aminotransferase gene in the genome might be involved in its biosynthesis or its synthesis proceeds partly via the mechanism described by Tryon et al. The analysis of the *S. lividans* genome, however, revealed a homolog of the XNR_1347 gene showing 87% identity. Thus, it could be that the product of this gene might also be involved in the biosynthesis of capreomycidine in *S. lividans*.

Comparisons of the cyclic hexapeptides encoding *flk* and mannopeptimycin gene cluster revealed a high degree of similarity. Both clusters exhibit some gene homologs that are involved in the biosynthesis and attachment of mannose, which suggests that both clusters originate from a common ancestor. Taking into account the similar core structures of the two peptide products, it might be interesting to use genes from both clusters, such as the isovaleryltransferase gene or the genes responsible for the biosynthesis and attachment of mannose, for combinatorial biosynthesis to generate unnatural analogs.

In summary, NPs with rare nonproteinogenic amino acid derivatives of arginine and their corresponding biosynthetic genes are of particular interest. They can serve as chemical scaffolds for semisynthetic modifications or as a natural library of genes for combinatorial biosynthesis to expand the structural diversity of nonribosomal peptides. In general, approaches based on searching for genes whose products are involved in the biosynthesis of interesting and unique moieties enable prioritization and targeted genome mining. This might represent a faster strategy for identifying new NPs with interesting structures and activities.

## Figures and Tables

**Table 1:** Strains and plasmids used in this study.

Bacterial Strains and Plasmids	Description	Source or Reference
*S. albus* J1074	Isoleucine and valine auxotrophic derivative of *S. albus* G (DSM 40313) lacking SalI-restriction activity	Salas J., Oviedo, Spain
*S. albus* del9	* S. albus * G (DSM 40313) lacking 9 secondary metabolite gene clusters	This work
*S. albus* del10	* S. albus * G (DSM 40313) lacking 10 secondary metabolite gene clusters	This work
*S. albus* del9 delXNR_1347	* S. albus * del9 strain carrying the deletion of the *XNR_1347* gene	This work
*E. coli* ET12567 (pUB307)	conjugative transfer of DNA	Kieser et al., 2000
*E. coli* GB05-red	Derivative of GB2005 containing integration of PBAD-ETgA operon	Zhang et al., 2000
pSMART	Chloramphenicol-resistant, general cloning BAC vector	Thermo Fisher Scientific
2K9/2	pSMART derivative containing a piece of the *S. albus* J1074 chromosome	Myronovskyi et al., 2018
2K9/2delXNR_1347	2K9/2 derivative containing deleted XNR_1347 gene	This work

**Table 2:** Primers used in this work.

Primers	Sequence 5′-3′	Purpose
XNR_1347RedFor	TGGAGCGGGAGCGCGAGCAGGACGAGCGGCGCGAGCGGGCGTGAGCCGCCGGGTGCCCGCGCCCGGTCATATCCATCCTTTTTCGCACGATATAC	XNR_1347 gene deletion
XNR_1347RedRev	CGACGCCTGGCTGCCGGGGCTCCCGCGCGGGTCCGTAGAATCGGCCCCACCATGGCCTACCTCGACCACCAGATTACGCGCAGAAAAAAAGGATCTC	
XNR_1347F	AAGAAGCAGCTGGAGCGGGAG	XNR_1347 gene deletion check
XNR_1347R	ACCATGGCCTACCTCGACCAC	

**Table 3:** Analysis of the adenylation domain specificity.

Domain	NRPS Signature Position of the Residues										Predicted Substrate
	235	236	239	278	299	301	322	330	331	517	
A_1_	D	A	W	T	V	A	A	V	C	K	Phe
A_2_	D	G	M	L	V	G	A	V	V	K	Leu
A_3_	D	A	F	W	L	G	G	T	F	K	Val
A_4_	D	L	A	E	S	G	A	V	D	K	Arg, Orn, Lys
A_5_	D	F	W	S	V	G	M	V	H	K	Thr
A_6_	D	F	W	S	V	G	M	V	H	K	Thr

## Supplementary Materials

The following are available online at https://www.mdpi.com/article/10.3390/microorganisms9081609/s1, Table S1. Comparison of the cyclofaulknamycin gene cluster from *S. albus* with the cluster from *S. kozangensis*; Table S2. NMR data of iso-faulknamycin 1 (DMSO-d6, 1H: 700 MHz, 13C: 175 MHz); Table S3. NMR data of cyclofaulknamycin 2 (DMSO-d6, 1H: 700 MHz, 13C: 175 MHz); Table S4. Stereoisomers obtained after the hydrolysis of capreomycin and chymostatin and the equivalent enantiomers with equivalent retention time (RT); Table S5. Calculated and observed y-ion and b-ion fragments of iso-faulknamycin; Table S6. Calculated and observed a-ion and x-ion fragments within 5 ppm of cyclofaulknamycin; Figure S1. The 1H NMR spectrum of iso-faulknamycin 1 (DMSO-d6, 700 MHz); Figure S2. COSY spectrum of iso-faulknamycin 1 (DMSO-d6, 700 MHz); Figure S3. HSQC spectrum of iso-faulknamycin 1 (DMSO-d6, 700 MHz, 175 MHz); Figure S4. HMBC spectrum of iso-faulknamycin 1 (DMSO-d6, 700 MHz, 175 MHz); Figure S5. ROESY spectrum of iso-faulknamycin 1 (DMSO-d6, 700 MHz); Figure S6. The 1H spectrum of cyclofaulknamycin 2 (DMSO-d6, 700 MHz); Figure S7. COSY spectrum of cyclofaulknamycin 2 (DMSO-d6, 700 MHz); Figure S8. HSQC spectrum of cyclofaulknamycin 2 (DMSO-d6, 700 MHz, 175 MHz); Figure S9. HMBC spectrum of cyclofaulknamycin 2 (DMSO-d6, 700 MHz, 175 MHz); Figure S10. ROESY spectrum of cyclofaulknamycin 2 (DMSO-d6, 700 MHz); Figure S11. Marfey’s chromatograms of the iso-faulknamycin hydrolysate (top) and the amino acid standards (bottom) derivatized with L-FDLA; Figure S12. Capreomycidine from capreomycin (3) and chymostatin (4); Figure S13. Marfey’s chromatograms showing the extracted mass of DLA-capreomycidine of the hydrolyzed L-DLA-iso-faulknamycin and the references obtained from the hydrolysis of capreomycidine and chymostatin derivatized with D-FDLA (DSR, DSS) and L-FDLA (LSS, LSR). DSR is equivalent to LRS; therefore, iso-faulknamycin contains the 2R 3S capreomycidine; Figure S14. D-threo-β-phenylserine (5) and L-threo-β-phenylserine (6); Figure S15. Marfey’s chromatograms showing the extracted mass of DLA-phenylserine of the hydrolyzed iso- faulknamycin and DL-threo-β-phenylserine, both derivatized with L-FDLA; Figure S16. Absolute configuration of iso-faulknamycin; Figure S17. Observed y-ion and b-ions from the MS/MS fragmentation (see Table S5) of iso-faulknamycin; Figure S18. MS/MS fragmentation spectrum of iso-faulknamycin ([M + H]^+^ peak); Figure S19. Observed a-ion and x-ions from the MS/MS fragmentation (see Table S6) of cyclofaulknamycin; Figure S20. MS/MS fragmentation spectrum of cyclofaulknamycin ([M + 2H]^2+^ peak).

## References

[1] Dias D.A., Urban S., Roessner U. (2012). A historical overview of natural products in drug discovery. Metabolites.

[2] Marcone G.L., Binda E., Berini F., Marinelli F. (2018). Old and new glycopeptide antibiotics: From product to gene and back in the post-genomic era. Biotechnol. Adv..

[3] Zhang D., Lu Y., Chen H., Wu C., Zhang H., Chen L., Chen X. (2020). Antifungal peptides produced by actinomycetes and their biological activities against plant diseases. J. Antibiot..

[4] Hudson G.A., Mitchell D.A. (2018). RiPP antibiotics: Biosynthesis and engineering potential. Curr. Opin. Microbiol..

[5] Strieker M., Tanović A., Marahiel M.A. (2010). Nonribosomal peptide synthetases: Structures and dynamics. Curr. Opin. Struct. Biol..

[6] Felnagle E.A., Jackson E.E., Chan Y.A., Podevels A.M., Berti A.D., McMahon M.D., Thomas M.G. (2008). Nonribosomal peptide synthetases involved in the production of medically relevant natural products. Mol. Pharm..

[7] Thomas M.G., Chan Y.A., Ozanick S.G. (2003). Deciphering tuberactinomycin biosynthesis: Isolation, sequencing, and annotation of the viomycin biosynthetic gene cluster. Antimicrob. Agents Chemother..

[8] Atkinson D.J., Naysmith B.J., Furkert D.P., Brimble M.A. (2016). Enduracididine, a rare amino acid component of peptide antibiotics: Natural products and synthesis. Beilstein J. Org. Chem..

[9] Petersen P.J., Wang T.Z., Dushin R.G., Bradford P.A. (2004). Comparative in vitro activities of AC98-6446, a novel semisynthetic glycopeptide derivative of the natural product mannopeptimycin, and other antimicrobial agents against gram-positive clinical isolates. Antimicrob. Agents Chemother..

[10] Magarvey N.A., Haltli B., He M., Greenstein M., Hucul J.A. (2006). Biosynthetic pathway for mannopeptimycins, lipoglycopeptide antibiotics active against drug-resistant gram-positive pathogens. Antimicrob. Agents Chemother..

[11] Yin X., Zabriskie T.M. (2006). The enduracidin biosynthetic gene cluster from *Streptomyces fungicidicus*. Microbiology.

[12] Guo C., Mandalapu D., Ji X., Gao J., Zhang Q. (2018). Chemistry and biology of teixobactin. Chemistry.

[13] Ling L.L., Schneider T., Peoples A.J., Spoering A.L., Engels I., Conlon B.P., Mueller A., Schäberle T.F., Hughes D.E., Epstein E. (2015). A new antibiotic kills pathogens without detectable resistance. Nature.

[14] Goldstein E., Eagle M.C., LaCasse M.L. (1971). In vitro chemotherapeutic combinations against isoniazid-resistant *Mycobacterium tuberculosis* and *Mycobacterium fortuitum*. Appl. Microbiol..

[15] Chan D.I., Prenner E.J., Vogel H.J. (2006). Tryptophan- and arginine-rich antimicrobial peptides: Structures and mechanisms of action. Biochim. Biophys. Acta.

[16] Kang J.H., Lee M.K., Kim K.L., Hahm K.S. (1996). Structure-biological activity relationships of 11-residue highly basic peptide segment of bovine lactoferrin. Int. J. Pept. Protein Res..

[17] Sikorska E., Stachurski O., Neubauer D., Małuch I., Wyrzykowski D., Bauer M., Brzozowski K., Kamysz W. (2018). Short arginine-rich lipopeptides: From self-assembly to antimicrobial activity. Biochim. Biophys. Acta Biomembr..

[18] Antonoplis A., Zang X., Wegner T., Wender P.A., Cegelski L. (2019). A vancomycin-arginine conjugate inhibits growth of carbapenem-resistant *E. coli* and targets cell-wall synthesis. ACS Chem. Biol..

[19] Myronovskyi M., Rosenkränzer B., Nadmid S., Pujic P., Normand P., Luzhetskyy A. (2018). Generation of a cluster-free *Streptomyces albus* chassis strains for improved heterologous expression of secondary metabolite clusters. Metab. Eng..

[20] Tryon J.H., Rote J.C., Chen L., Robey M.T., Vega M.M., Phua W.C., Metcalf W.M., Ju K.-S., Kelleher N.L., Thomson R.J. (2020). Genome mining and metabolomics uncover a rare d-capreomycidine containing natural product and its biosynthetic gene cluster. ACS Chem. Biol..

[21] Fu J., Wenzel S.C., Perlova O., Wang J., Gross F., Tang Z., Yin Y., Stewart A.F., Müller R., Zhang Y. (2008). Efficient transfer of two large secondary metabolite pathway gene clusters into heterologous hosts by transposition. Nucleic Acids Res..

[22] Zhang Y., Muyrers J.P.P., Testa G., Stewart A.F. (2000). DNA cloning by homologous recombination in *Escherichia coli*. Nat. Biotechnol..

[23] Kieser T., Bibb M.J., Buttner M.J., Chater K.F., Hopwood D.A. (2000). Practical Streptomyces Genetics.

[24] Sambrook J., Russell D. (2001). Molecular Cloning: A Laboratory Manual.

[25] Blin K., Shaw S., Kloosterman A.M., Charlop-Powers Y., van Wezel G.P. (2021). antiSMASH 6.0: Improving cluster detection and comparison capabilities. Nucleic Acids Res..

[26] Yin X., McPhail K.L., Kim K.J., Zabriskie T.M. (2004). Formation of the nonproteinogenic amino acid 2S,3R-capreomycidine by VioD from the viomycin biosynthesis pathway. ChemBioChem.

[27] Kohli R.M., Walsh C.T. (2003). Enzymology of acyl chain macrocyclization in natural product biosynthesis. Chem. Commun..

[28] Baltz R.H. (2011). Function of MbtH homologs in nonribosomal peptide biosynthesis and applications in secondary metabolite discovery. J. Ind. Microbiol. Biotechnol..

[29] Hackl S., Bechthold A. (2015). The gene *bldA*, a regulator of morphological differentiation and antibiotic production in streptomyces. Arch. Pharm..

[30] Harada K., Fujii K., Hayashi K., Suzuki M., Ikai Y., Oka H. (1996). Application of d,l-FDLA derivatization to determination of absolute configuration of constituent amino acids in peptide by advanced Marfey’s method. Tetrahedron Lett..

[31] Rebets Y., Broetz E., Tokovenko B., Luzhetskyy A. (2014). Actinomycetes biosynthetic potential: How to bridge in silico and in vivo?. J. Ind. Microbiol. Biotechnol..

[32] Belknap K.C., Park C.J., Barth B.M., Andam C.P. (2020). Genome mining of biosynthetic and chemotherapeutic gene clusters in *Streptomyces* bacteria. Sci. Rep..

