# Supplementary material for: Cyclofaulknamycin with the Rare Amino Acid D-capreomycidine Isolated from a Well-Characterized Streptomyces albus Strain
Source: Microorganisms. 2021 Jul 28;9(8):1609. doi: 10.3390/microorganisms9081609 (PMC8399532; doi:10.3390/microorganisms9081609)
Supplement: Supplementary file 1 [file microorganisms-09-01609-s001.zip › microorganisms-1279128-supplementary.pdf]

Supplementary Materials

Cyclofaulknamycin with the rare amino acid D-capreomycin isolated from a well-characterised *Streptomyces albus* strain

Horbal L.[1,3§], Stierhof M.[1§], Palusczak A.[1], Eckert N[1]., Zapp. J., Luzhetskyy A.[1,2*]

Department of Pharmaceutical Biotechnology. Saarland University, 66123 Saarbruecken, Germany, Lihorbal@gmail.com (L.H.); marc.stierhof@uni-saarland.de (M.S.); anja.palusczak@uni-saarland.de (P.A.)

2 Department of Pharmaceutical Biology. Saarland University, 66123 Saarbruecken, Germany; j.zapp@mx.uni-saarland.de (J.Z.)

3 AMEG Department , Helmholtz Institute for Pharmaceutical Research Saarland, 66123 Saarbruecken Germany, nikolas.eckert@uni-saarland.de (N.E.)

* Correspondence: a.luzhetskyy@mx.uni-saarland.de; Tel.: +49-681-302-70200 (A.L.)

Table S1. Comparison of the cyclofaulknamycin gene cluster from *S. albus* with the cluster from *S. kozangensis*.

Gene in the *S. albus* genome	Gene name	Putative product	Locus in the genome of S. *koyangensis* VK-A60T
XNR_0965		urea ABC transporter permease subunit UrtC [Streptomyces albidoflavus]	
XNR_0966		urea ABC transporter ATP-binding protein UrtD [Streptomyces albidoflavus]	
XNR_0967		ATP-binding cassette domain-containing protein [Streptomyces albidoflavus]	
XNR_0968	*flk*A	DeoR/GlpR transcriptional regulator [Streptomyces sp. CS227]	D0C37_RS06225
XNR_0969	*flk*B	sugar ABC transporter substrate-binding protein [Streptomyces albidoflavus]	D0C37_RS06230
XNR_0970	*flk*C	integral membrane sugar transporter	D0C37_RS06235
XNR_0971	*flk*D	carbohydrate ABC transporter permease	D0C37_RS06240
XNR_0972	*flk*E	zinc-dependent alcohol dehydrogenase family protein	D0C37_RS06245
XNR_0973	*flk*F	DUF2029 domain-containing protein	D0C37_RS06250
XNR_0974	*flk*G	FkbM family methyltransferase	D0C37_RS06255
XNR_0975	*flk*H	glycosyltransferase family 4 protein alpha-(1-2)-phosphatidylinositol mannosyltransferase	D0C37_RS06260
XNR_0976	*flk*I	response regulator transcription factor	D0C37_RS06265
XNR_0977	*flk*J	histidine kinase	D0C37_RS06270
XNR_0978	*flk*K	ABC transporter ATP-binding protein	D0C37_RS06275
XNR_0979	*flkL*	ABC type antimicrobial transporter permease, FtsX-like permease family protein	D0C37_RS06280
XNR_0980	*flkM*	LuxR family response regulator	D0C37_RS06285
XNR_0981	*flkN*	Two component sensor histidine kinase	D0C37_RS06290
XNR_0982	*flkO*	Alpha/beta hydrolase MppK beta-lactamase, serine hydrolase	D0C37_RS06295
XNR_0983	*flkP*	NRPS	D0C37_RS06300
XNR_0984	*flkR*	MbtH family protein	D0C37_RS06305
XNR_0985	*flkS*	NRPS	D0C37_RS06310
-	-	NRPS	D0C37_RS06315
XNR_0986	*flkT*	Fe(II)/alpha-ketoglutarate-dependent arginine beta-hydroxylase	D0C37_RS06320
XNR_0987	*flkU*	Major Facilitator Superfamily transporter	D0C37_RS06325
XNR_0988	*flkV*	Cytochrome 450	D0C37_RS06330
XNR_0989	*flkW*	ArsR family transcriptional regulator	D0C37_RS06335
XNR_0990	*flkX*	Thioeterase, alpha|beta hydrolase	D0C37_RS06340
XNR_0991		Erythromycin esterase protein	
XNR_0992		class I SAM-dependent RNA methyltransferase	
XNR_0993		phenylalanine-specific permease	
XNR_0994		TrkA family potassium uptake protein	
XNR_0995		TrkA family potassium uptake protein	
XNR_0996		DUF3159 domain-containing protein	
XNR_0997		OB-fold nucleic acid binding domain-containing protein	
XNR_0998		Osmosensitive K+ channel histidine kinase KdpD, response regulator	
XNR_0999		sensor histidine kinase KdpD	
XNR_1000		ABC transporter	
XNR_1001		Short chain dehydrogenase	
XNR_1002		TetR/AcrR family transcriptional regulator	
XNR_1003		DUF3710 domain-containing protein	
XNR_1347		PLP-dependent aminotransferase (pyrodoxal dependent)	

1. NMR Spectroscopy

Table S2. NMR data of iso-faulknamycin **1** (DMSO-d_6, ^{1}H: 700 MHz, ^{13}C: 175 MHz)

unit	δC	δH, multiplicity, (J in Hz)	COSY	ROESY	HMBC (H-)
1 – COOH	n.a.[1]				
2 – CH	58.1	4.22, m	3, 5		
3 – CH	66.4	4.12, m	2, 4		
4 – CH_3	20.3	1.06, d (6.1)	3		2, 3
5 – NH		8.37	2		
6 – CO	n.a.[1]				
7 – CH	54.3	4.70, ovl.[2]	8, 14		
8 – CH	50.2	3.73, m	7, 9		8
9 – CH_2a<br>CH_2b	22.0	1.93, ovl.[2]<br>1.67, m	8, 10		
10 – CH_2a<br>CH_2b	36.1	3.37, ovl.[2]<br>3.19, m	9, 11		
11 – NH		8.09, bs	10		
12 – C		n.a.[1]			
12 – NH		n.a.[1]			
13 – NH		n.a.[1]			
14 – NH		8,41, d (8.2)	7	16	
15 – CO	171.3				
16 – CH	57.8	4.33, t (8.1)	17, 20	14	15, 21
17 – CH	30.9	2.01, m	16, 18, 19		
18 – CH_3	19.3	0.84, ovl.[2]	17		16 ,17 ,19
19 – CH_3	18.1	0.82, ovl.[2]	17		16 ,17 ,18
20 – NH		8.24, bs	16	22	
21 – CO	172.1				
22 – CH	51.2	4.52, m	23 ,27	20	
23 – CH_2a<br>CH_2b	41.8	1.49, ovl.[2]<br>1.40, m	22, 24		
24 – CH	24.2	1.50, ovl.[2]	23, 25, 26		
25 – CH_3	23.2	0.86, ovl.[2]	24		23 ,24 ,26
26 – CH_3	21.6	0.81, ovl.[2]	24		23 ,24 ,25
27 – NH		8.27, d (8.4)	22		28
28 – CO	169.0				
29 – CH	58.7	4.72, ovl.[2]	30 ,37		28
30 – CH	72.8	5.11, m	29, 30-OH		31, 32/36
30 – OH		5.68, d (4.6)	30		29, 30
31 – C	142.1				
32/36 – CH	126.1	7.42, d (7.3)	33/35		30, 32, 34, 36
33/35 – CH	127.6	7.27, t (7.5)	32/36, 34		31, 33, 35
34 – CH	127.0	7.20, t (7.0)	33/35		32, 36
37 – NH		8.69, d (8.7)	29	39	
38 – CO	n.a.[1]				
39 – CH	57.4	3.93, m	40, 42	37	
40 – CH	64.6	3.83, m	39, 41, 40 OH		
40 – OH		5.39, bs	40		
41 – CH3	16.5	0.39, d (6.0)	40		39, 40
42 – NH2		7.88, bs	39		

[1]n.a. = not available; [2]ovl. = signal overlap

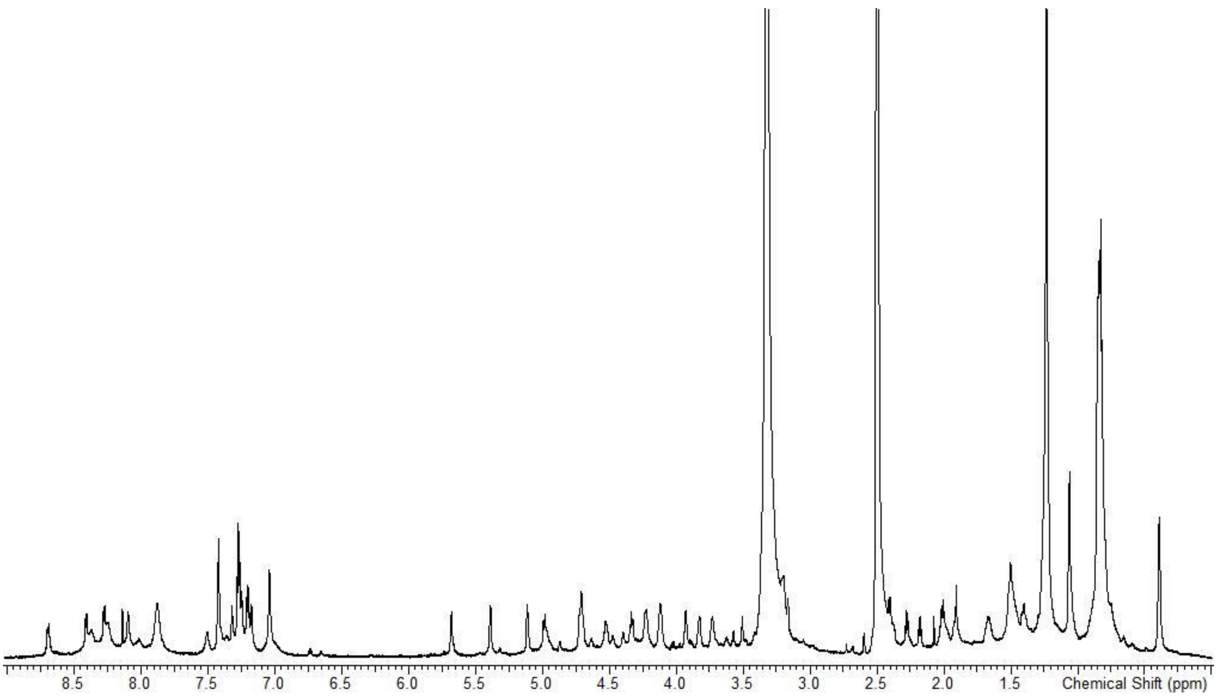

Figure S1: ^{1}H NMR spectrum of iso-faulknamycin **1** (DMSO-d6, 700 MHz)

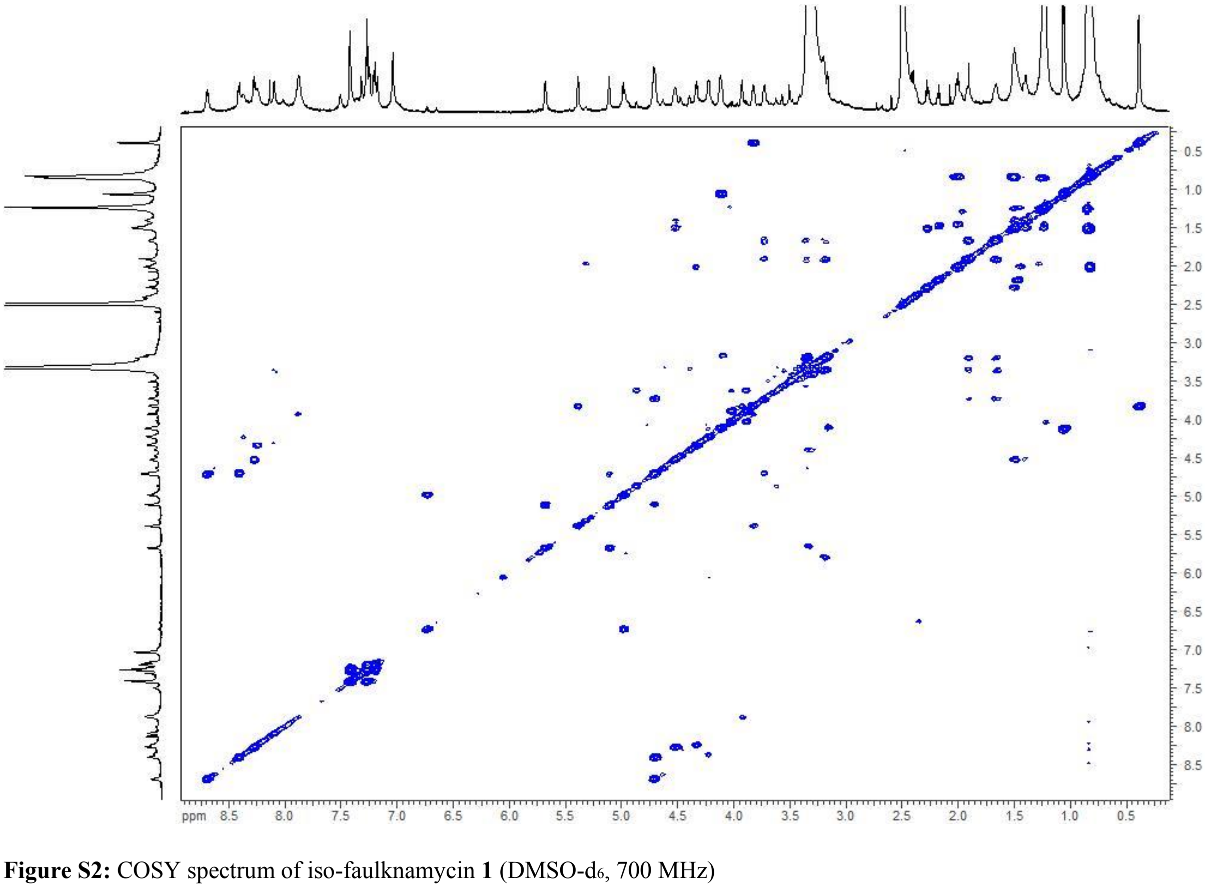

Figure S2: COSY spectrum of iso-faulknamycin **1** (DMSO-d6, 700 MHz)

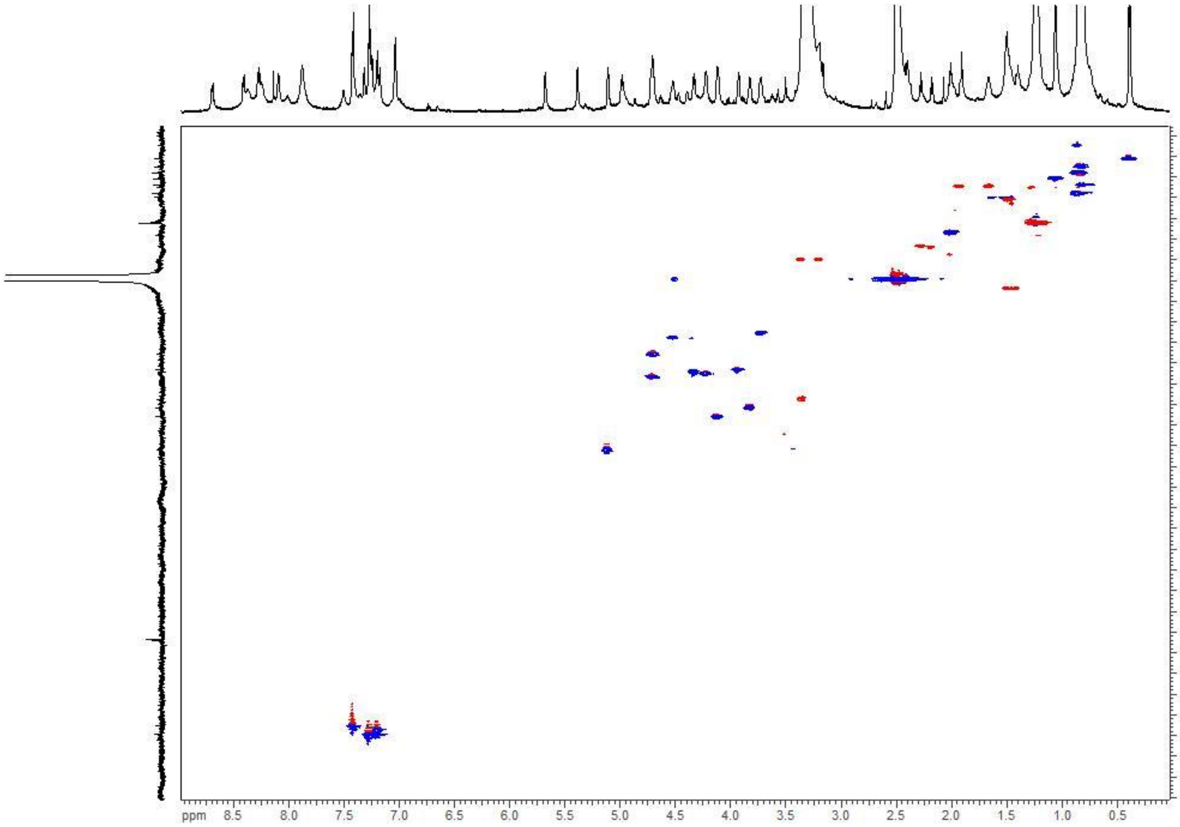

Figure S3: HSQC spectrum of iso-faulknamycin **1** (DMSO-d6, 700 MHz, 175 MHz)

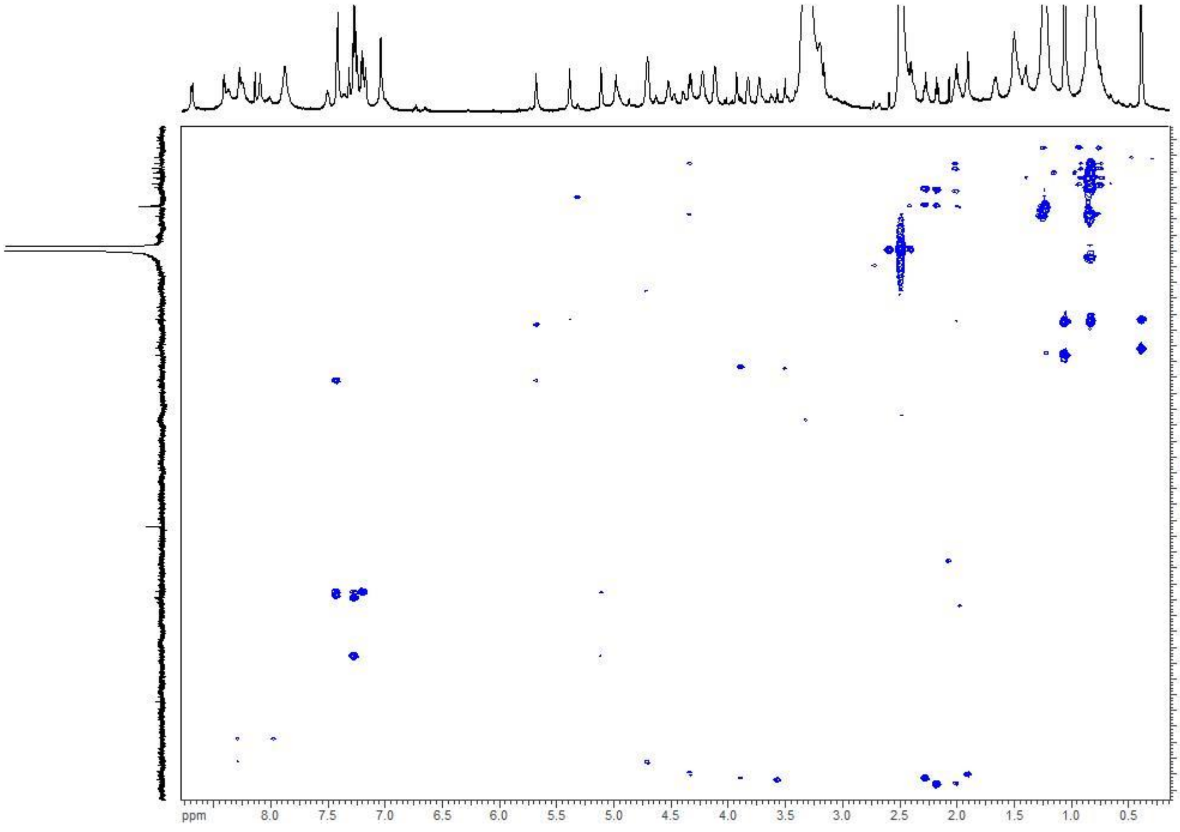

Figure S4: HMBC spectrum of iso-faulknamycin **1** (DMSO-d6, 700 MHz, 175 MHz)

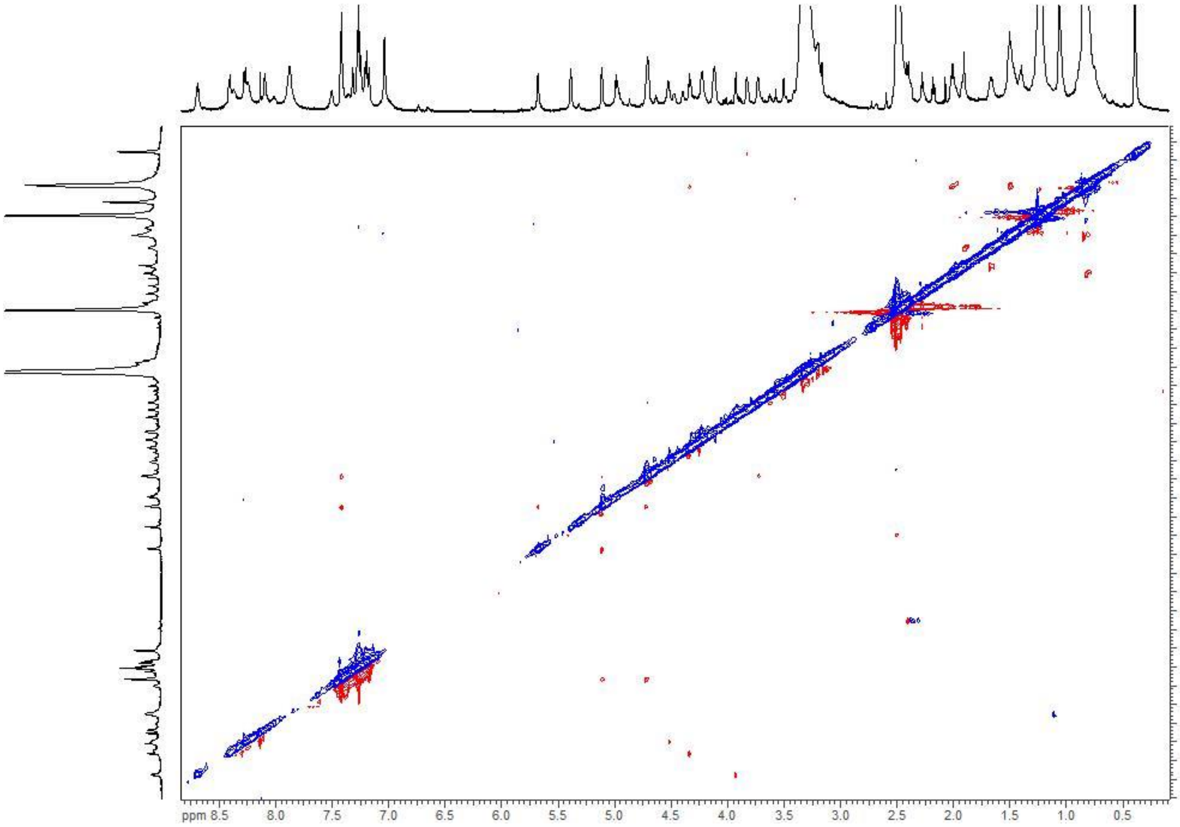

Figure S5: ROESY spectrum of iso-faulknamycin **1** (DMSO-d_6, 700 MHz)

Table S3: NMR data of cyclofaulknamycin **2** (DMSO-d_6, ^{1}H: 700 MHz, ^{13}C: 175 MHz)

unit	δ_C	δ_H, multiplicity, (J in Hz)	COSY	ROESY	HMBC (H-)
1 – CO	171.6				
2 – CH	62.7	3.89, m	3, 5	42	
3 – CH	65.0	3.85, ovl	2, 4		
4 – CH_3	20.9	1.09, d (6.0)	3		2, 3
5 – NH		8.94, bs	2		
6 – CO	n.a				
7 – CH	54.5	4.69, t (6.6)	8, 14		
8 – CH	50.7	3.84, ovl	7, 9		
9 – CH_2	21.3	1.76, m	8, 10		
10 – CH_2	36.3	3.11	9		
11 – NH		n.a			
12 – C	n.a				
12 – NH		n.a.			
13 – NH		n.a			
14 – NH		7.91, bs	7		
15 – CO	n.a				
16 – CH	60.1	3.97, dd (6.4, 11.6)	17, 20		21
17 – CH	29.4	2.2, m	16 ,18 ,19		
18 – CH_3	20.0	0.86, ovl	17		16 ,17 ,19
19 – CH_3	18.5	0.88, ovl	17		16 ,17 ,18
20 – NH		8.73	16	22	
21 – CO	172.4				
22 – CH	52.3	4.34, m	23 ,27	20	
23 – CH_2a<br>CH_2b	40.4	1.37, ovl<br>1.46, m	22, 24		21
24 – CH	24.9	1.38, ovl	23 ,25 ,26		21
25 – CH_3	23.1	0.79, d (5.9)	24		23, 24 ,26
26 – CH_3	23.1	0.87, ovl	24		23 ,24 ,25
27 – NH		8.01, ovl	22	29	28
28 – CO	169.7				
29 – CH	60.0	4.58, dd (8.2, 3.9)	30, 37	27	28
30 – CH	73.3	5.03, d (3.0)	29		32, 36
31 – C	142.3				
32/36 – CH	127.2	7.29, d (7.1)	33/35		30, 32/36, 34
33/35 – CH	128.1	7.25, t (7.2)	32/36, 34		31, 33/35
34 – CH	127.5	7.20, t (7.1)	33/35		32, 36
37 – NH		7.64, bs	29	39	
38 – CO	n.a				
39 – CH	57.7	4.16, t (7.3)	40, 42	37	1
40 – CH	66.0	3.93, m	39, 41		
41 – CH_3	20	0.98, d (6.2)	40		39, 40
42 – NH		8.52, ovl	39	2	

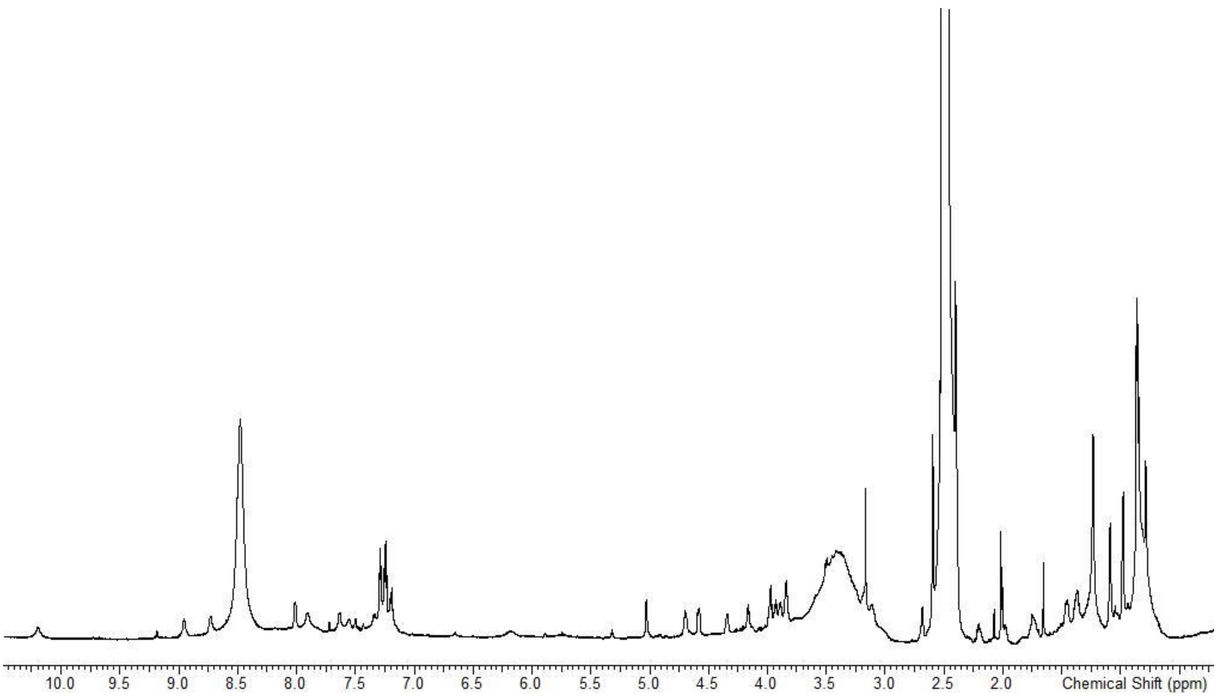

Figure S6: ^{1}H spectrum of cyclofaulknamycin **2** (DMSO-d_6, 700 MHz)

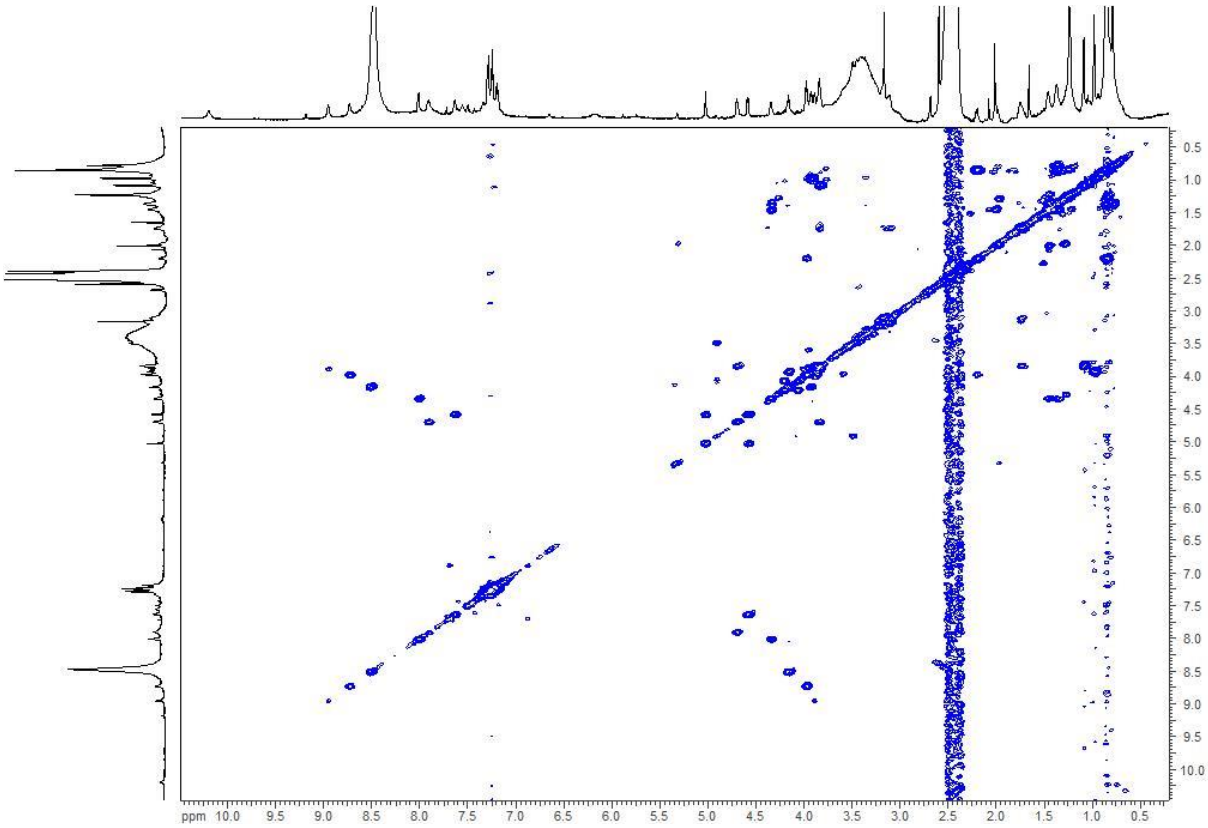

Figure S7: COSY spectrum of cyclofaulknamycin **2** (DMSO-d_6, 700 MHz)

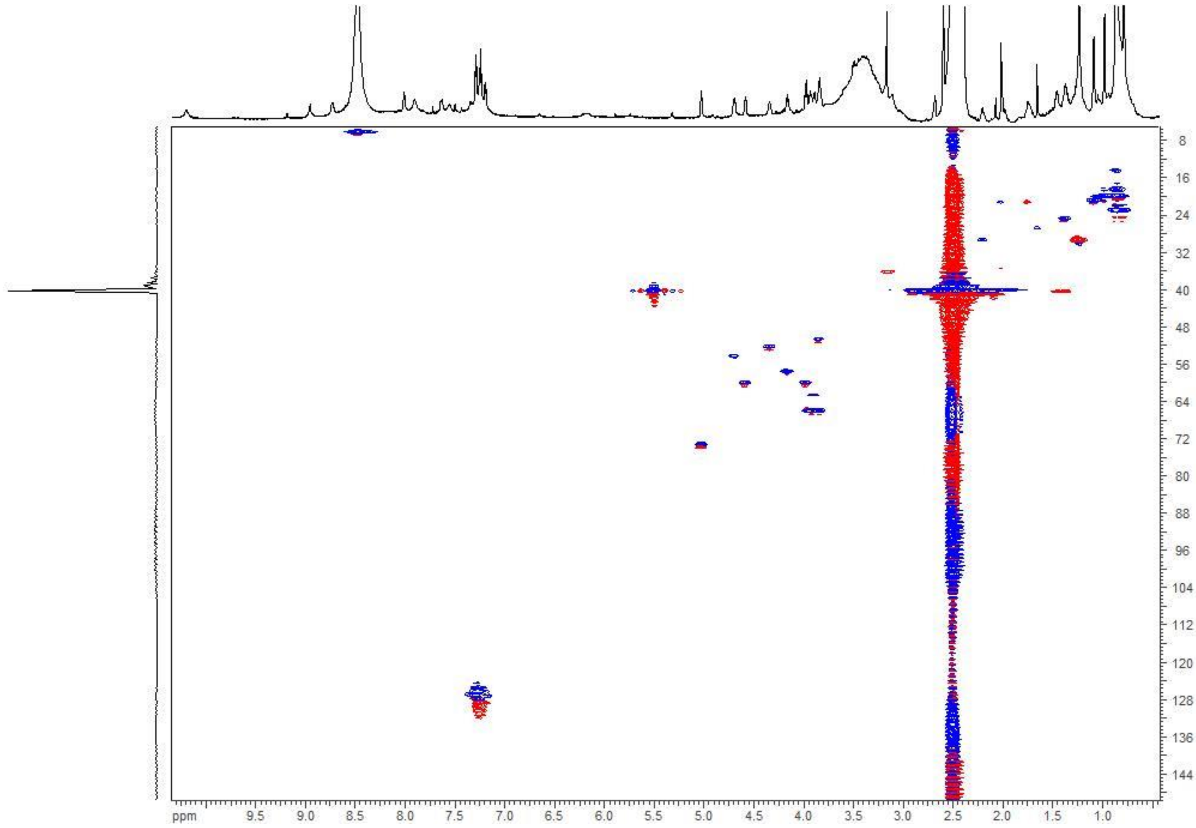

Figure S8: HSQC spectrum of cyclofaulknamycin **2** (DMSO-d6, 700 MHz, 175 MHz)

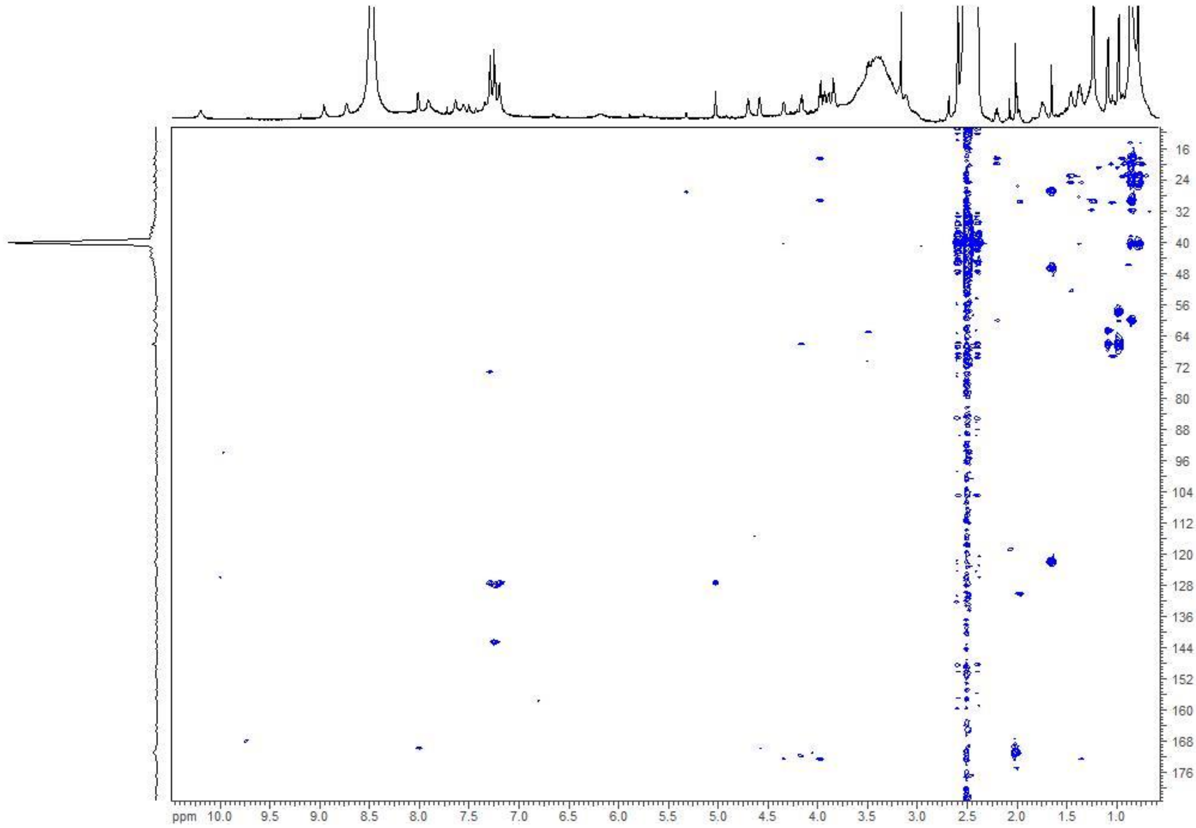

Figure S9: HMBC spectrum of cyclofaulknamycin **2** (DMSO-d6, 700 MHz, 175 MHz)

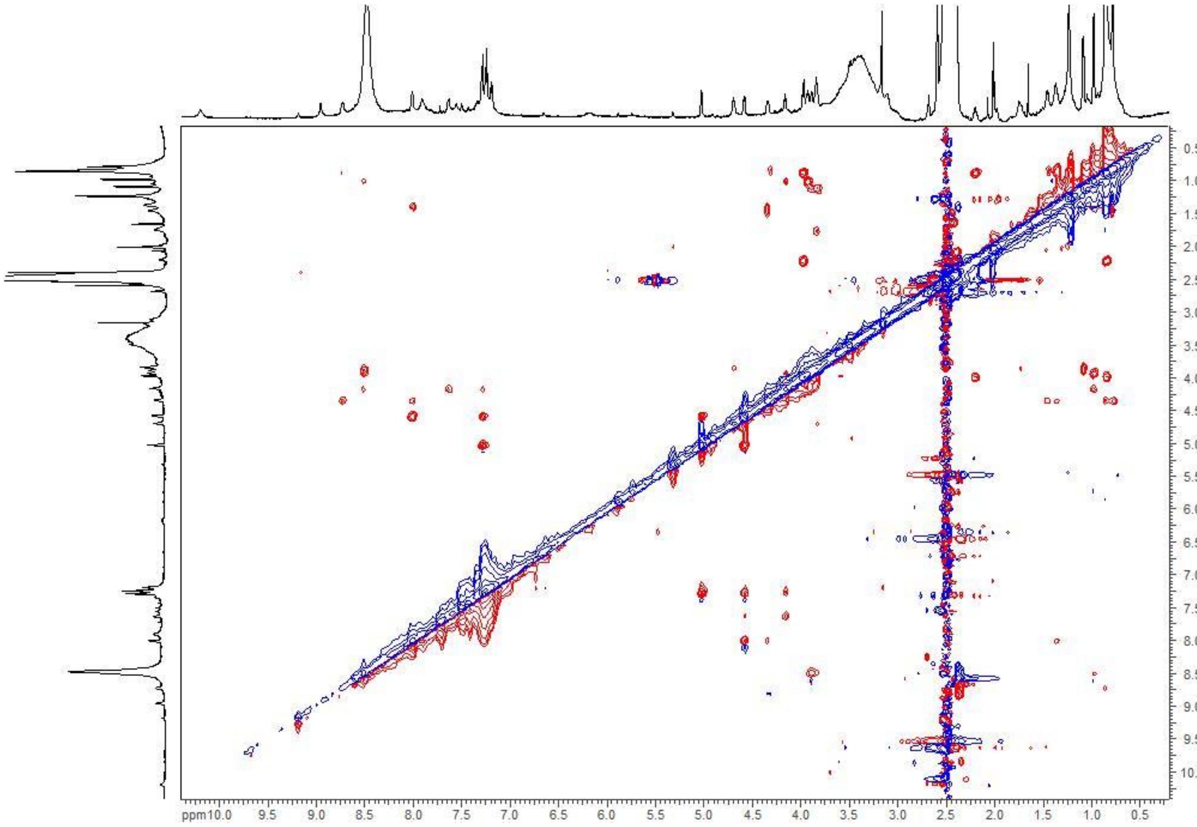

Figure S1: ROESY spectrum of cyclofaulknamycin **2** (DMSO-d6, 700 MHz)

2. Marfey's Analysis

Standards in D- and L-configuration were derivatized with L-FDLA and compared with the amino acids derived from hydrolysis of **1** (Fig. S11).

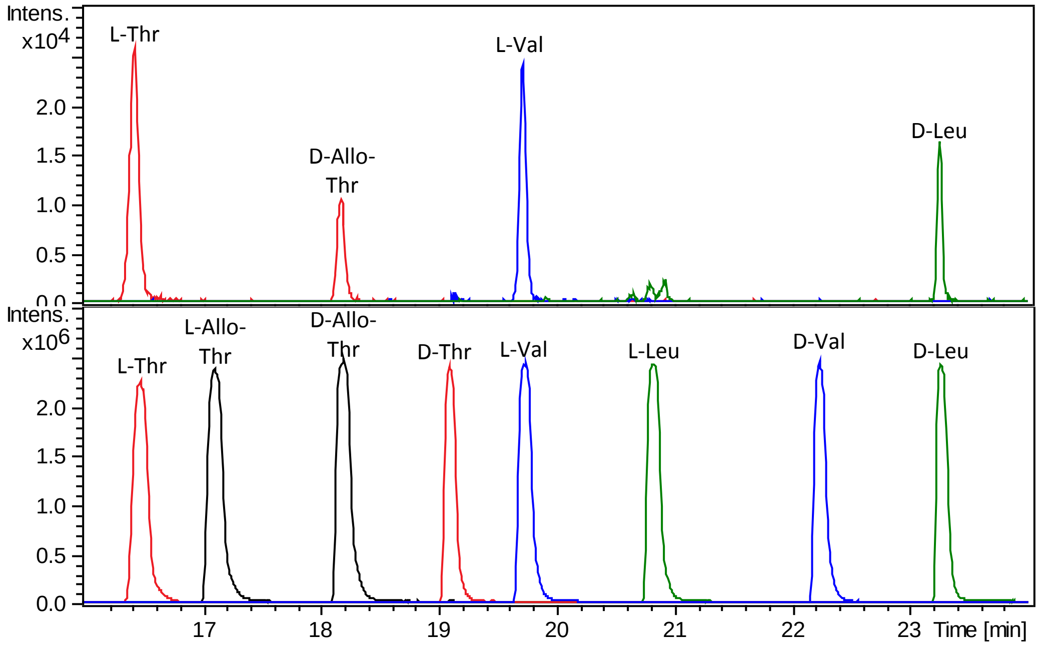

Figure S2: Marfey's chromatograms of the iso-faulknamycin hydrolysate (top) and the amino acids standards (bottom) derivatized with L-FDLA.

We obtained 2S-3R-capreomycidine **3** and 2S-3S-capreomycidine **4** by hydrolysis of capreomycin and chymostatin, respectively (fig. S12). The hydrolysates were derivatized with L-FDLA and D-FDLA, yielding all possible enantiomers with distinguishable retention time (table S4).

Table S4: Stereoisomers obtained after hydrolysis of capreomycin and chymostatin and the equivalent enantiomers with equivalent retention time (RT).

hyd. of capreomycin	L-FDLA	D-FDLA	hyd. of chymostatin	L-FDLA	D-FDLA
2S-3R-capreomycidine	*LSR*	*DSR*	2S-3S-capreomycidine	*LSS*	*DSS*
RT equivalent to	*DRS*	*LRS*	RT equivalent to	*DRR*	*LRR*

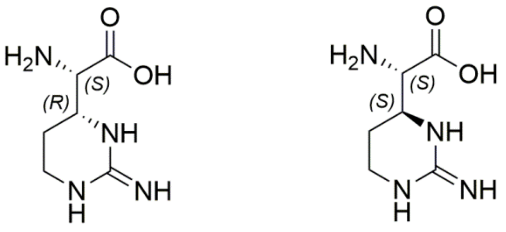

3 **4**

Figure S12: Capreomycidine from capreomycine (**3**) and chymostatine (**4**).

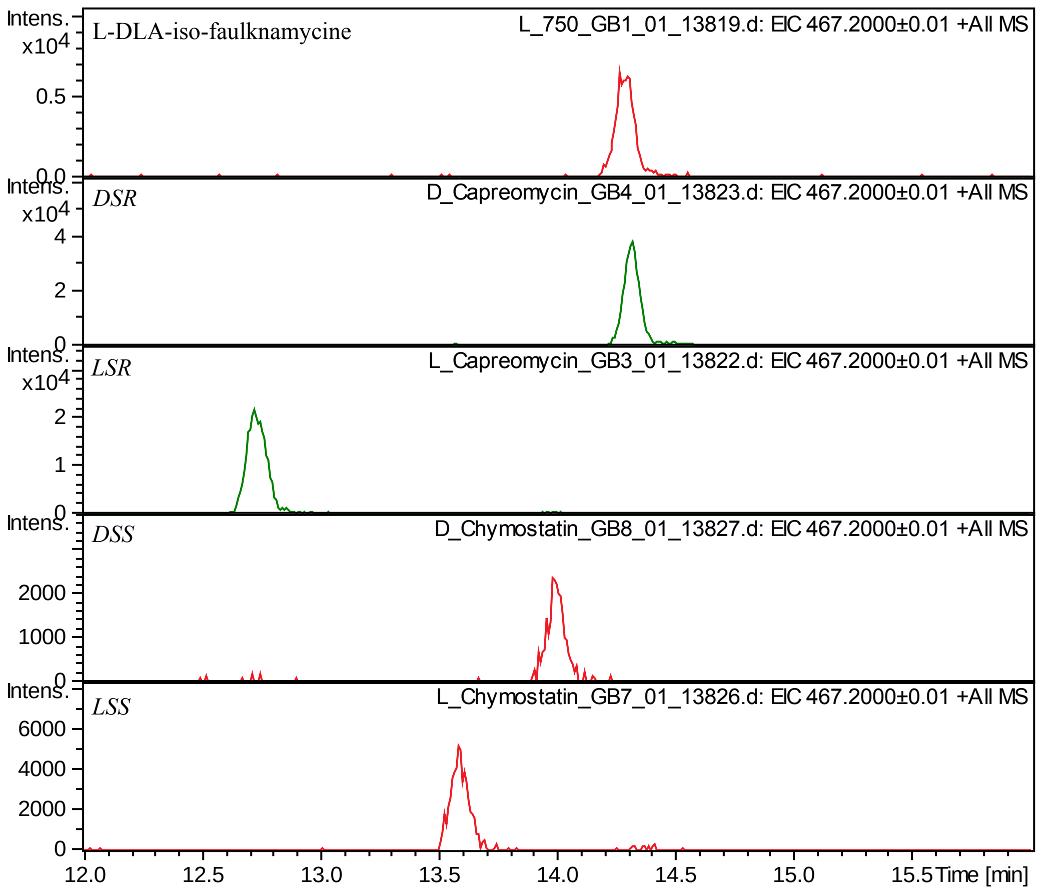

Figure S13: Marfey's chromatograms showing the extracted mass of DLA-capreomycidine of the hydrolysed L-DLA-iso-faulknamycin and the references obtained from hydrolysis of capreomycidine and chymostatin derivatized with D-FDLA (DSR, DSS) and L-FDLA (LSS, LSR). DSR is equivalent to LRS, therefore iso-faulknamycin contains the 2R-3S-capreomycidine.

D-*threo*-β-phenylserine **5** has been determined to be the constituent amino acid in faulknamycin (fig. S14). Therefore, we used DL-*threo*-β-phenylserine as a reference to determine the stereochemistry of β-phenylserine of the hydrolysed iso-faulknamycin (Fig. S14, S16).

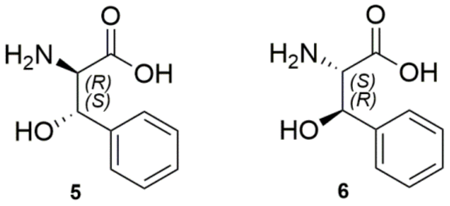

Figure S14: D-*threo*-β-phenylserine (**5**) and L-*threo*-β-phenylserine (**6**)

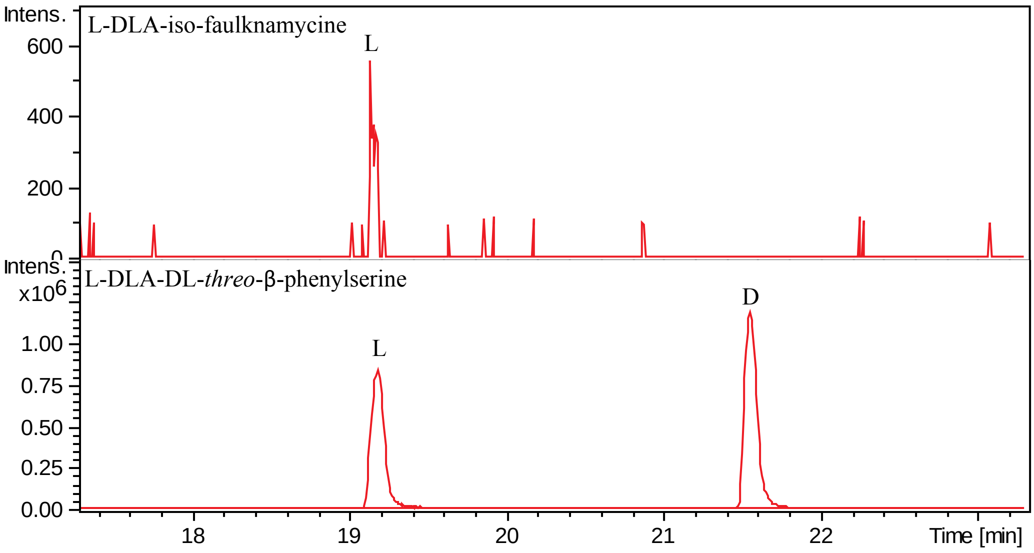

Figure S15: Marfey's chromatograms showing the extracted mass of DLA-phenylserine of the hydrolysed iso-faulknamycin and DL-*threo*-β-phenylserine, both derivatized with L-FDLA.

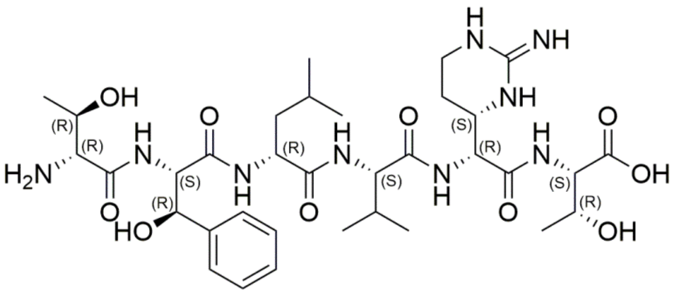

Figure S16: Absolute configuration of iso-faulknamycin.

3. MS/MS Fragmentation

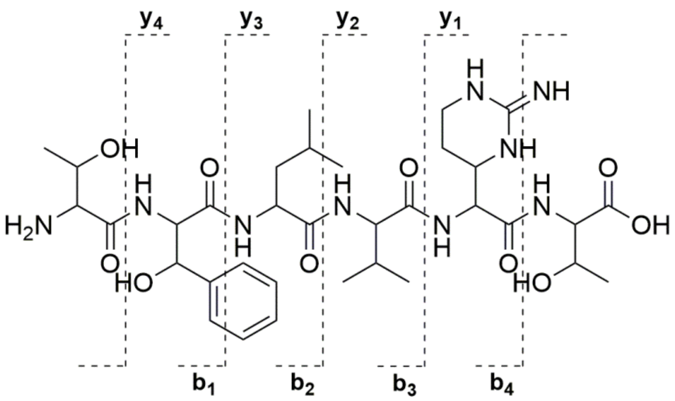

Figure S3: Observed y-ion and b-ions from the MS/MS fragmentation (see table 5) of iso-faulknamycin

Table S5: Calculated and observed y-ion and b-ion fragments of iso-faulknamycin.

ion type	y_1	y_2	y_3	y_4
[M]+ calculated	274.1511	373.2190	486.3032	649.3675
[M]+ observed	274.1517	373.2180	486.3034	649.3674
Δ [ppm]	-2.2	3.8	-0.4	0.2
**ion type**	b_1	b_2	b_3	b_4
[M]+ calculated	265.1186	378.2019	477.2697	631.3556
[M]+ observed	265.1185	378.2021	477.2698	631.3573
Δ [ppm]	0.4	-0.5	-0.2	-2.7

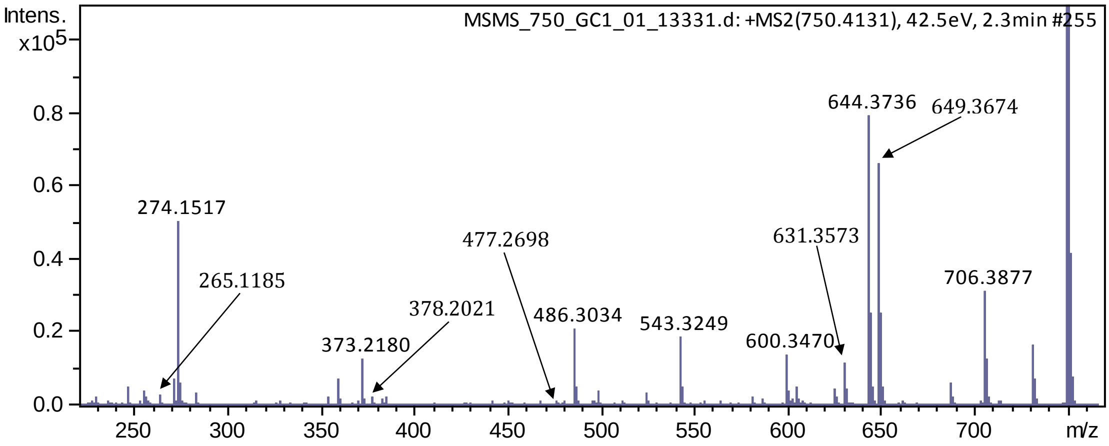

Figure S18: MS/MS fragmentation spectrum of iso-faulknamycin ($[M+H]^+$ peak).

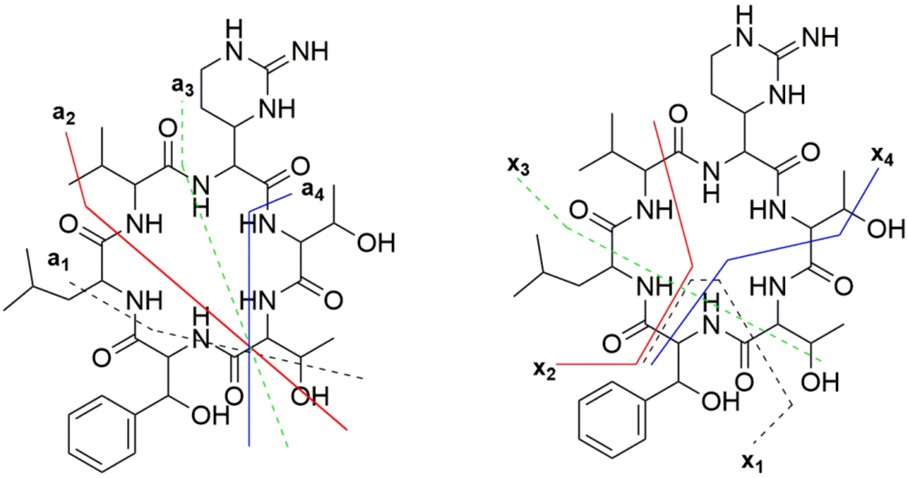

Figure S19: Observed a-ion and x-ions from the MS/MS fragmentation (see table 6) of cyclofaulknamycin

Table S6: Calculated and observed a-ion and x-ion fragments within 5 ppm of cyclofaulknamycin.

**ion type**	a_1	a_2	a_3	a_4
**AA sequence**	T_1-T_2	T_1-T_2-Cmp	T_1-T_2-Cmp-V	T_1-T_2-Cmp-V-L
**[M]+ calculated**	175.1077	329.1932	428.2616	541.3457
**[M]+ observed**	175.1082	329.1940	428.2622	541.3474
**deviation [ppm]**	2.9	2.4	1.4	3.1
**ion type**	x_1	x_2	x_3	x_4
**AA sequence**	βPhS(-H_2O)	L-V	V-cmp-T_2-T_1(-H_2O)	L-V-Cmp-T_2(-H_2O)
**[M]+ calculated**	146.0600	211.1441	438.2459	466.2772
**[M]+ observed**	146.0607	211.1444	438.2470	466.2782
**deviation [ppm]**	4.8	1.4	2.5	2.1

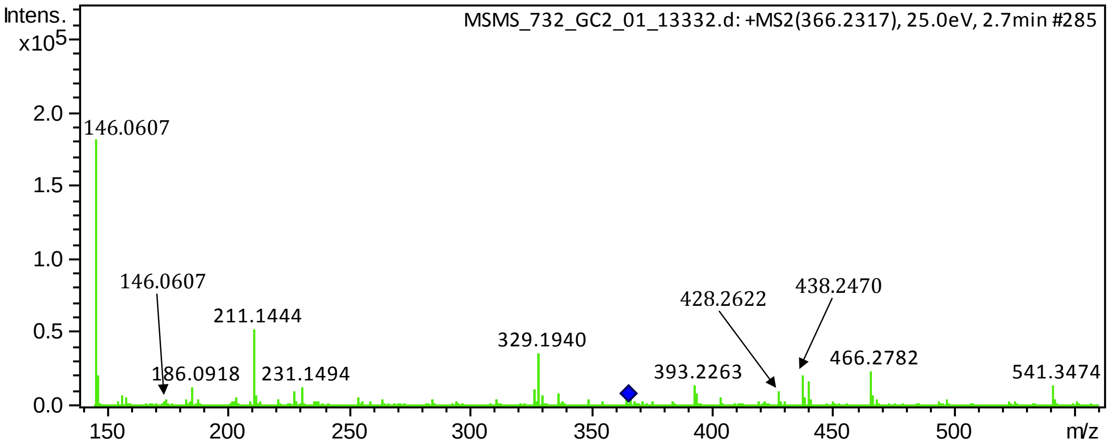

Figure S20: MS/MS fragmentation spectrum of cyclofaulknamycin ($[M+2H]^{2+}$ peak)

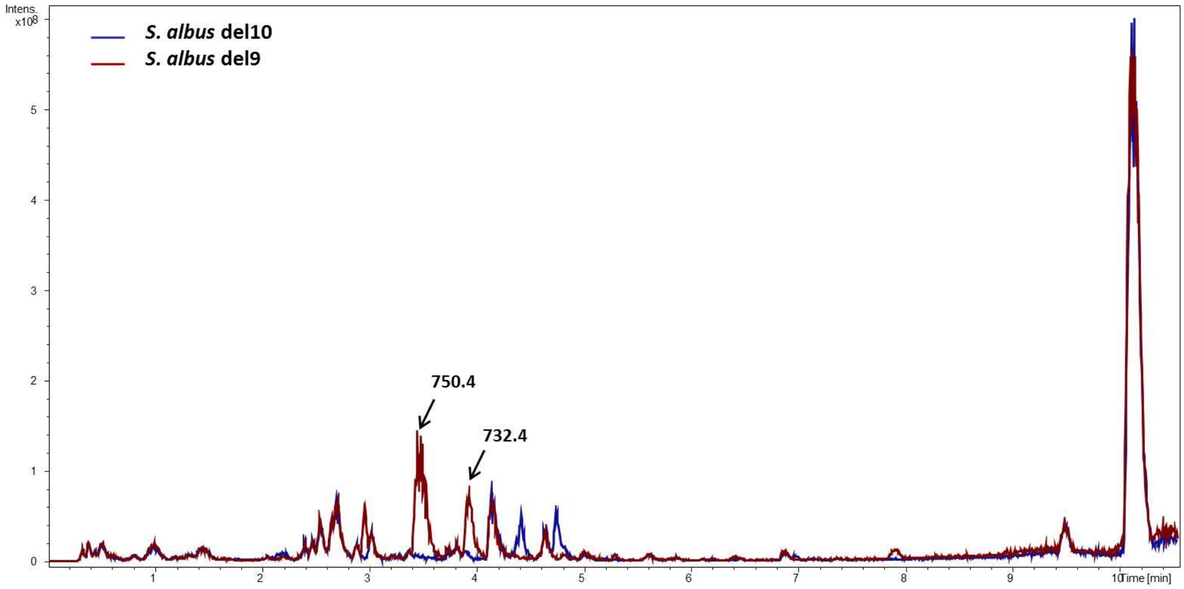

Figure S21: Secondary metabolite profile of the *S. albus* del9 and del 10 strains. Butanol extraction.
